# Factor Xa cleaves SARS-CoV-2 spike protein to block viral entry and infection

**DOI:** 10.1038/s41467-023-37336-9

**Published:** 2023-04-06

**Authors:** Wenjuan Dong, Jing Wang, Lei Tian, Jianying Zhang, Erik W. Settles, Chao Qin, Daniel R. Steinken-Kollath, Ashley N. Itogawa, Kimberly R. Celona, Jinhee Yi, Mitchell Bryant, Heather Mead, Sierra A. Jaramillo, Hongjia Lu, Aimin Li, Ross E. Zumwalt, Sanjeet Dadwal, Pinghui Feng, Weiming Yuan, Sean P. J. Whelan, Paul S. Keim, Bridget Marie Barker, Michael A. Caligiuri, Jianhua Yu

**Affiliations:** 1 Department of Hematology & Hematopoietic Cell Transplantation, City of Hope National Medical Center, Los Angeles, CA 91010 USA; 2 Hematologic Malignancies Research Institute, City of Hope National Medical Center, Los Angeles, CA 91010 USA; 3 Department of Computational and Quantitative Medicine, City of Hope National Medical Center, Los Angeles, CA 91010 USA; 4 Pathogen and Microbiome Institute, Northern Arizona University, Flagstaff, AZ 86011 USA; 5 Department of Biological Sciences, Northern Arizona University, Flagstaff, AZ 86011 USA; 6 Section of Infection and Immunity, Herman Ostrow School of Dentistry, Norris Comprehensive Cancer Center, University of Southern California, Los Angeles, CA 90089 USA; 7 Department of Molecular Microbiology and Immunology, Keck School of Medicine of University of Southern California, Los Angeles, CA 90033 USA; 8 Pathology Core of Shared Resources Core, Beckman Research Institute, City of Hope National Medical Center, Los Angeles, CA 91010 USA; 9 Department of Pathology, University of New Mexico, Albuquerque, NM 87131 USA; 10 Division of Infectious Diseases, Department of Medicine, City of Hope National Medical Center, Los Angeles, CA 91010 USA; 11 Department of Molecular Microbiology, Washington University School of Medicine, St. Louis, MO 63110 USA; 12 City of Hope Comprehensive Cancer Center, Los Angeles, CA 91010 USA; 13 Department of Immuno-Oncology, City of Hope, Los Angeles, CA 91010 USA

**Keywords:** SARS-CoV-2, Viral host response, Viral infection

## Abstract

Serine proteases (SP), including furin, trypsin, and TMPRSS2 cleave the SARS-CoV-2 spike (S) protein, enabling the virus to enter cells. Here, we show that factor (F) Xa, an SP involved in blood coagulation, is upregulated in COVID-19 patients. In contrast to other SPs, FXa exerts antiviral activity. Mechanistically, FXa cleaves S protein, preventing its binding to ACE2, and thus blocking viral entry and infection. However, FXa is less effective against variants carrying the D614G mutation common in all pandemic variants. The anticoagulant rivaroxaban, a direct FXa inhibitor, inhibits FXa-mediated S protein cleavage and facilitates viral entry, whereas the indirect FXa inhibitor fondaparinux does not. In the lethal SARS-CoV-2 K18-hACE2 model, FXa prolongs survival yet its combination with rivaroxaban but not fondaparinux abrogates that protection. These results identify both a previously unknown function for FXa and an associated antiviral host defense mechanism against SARS-CoV-2 and suggest caution in considering direct FXa inhibitors for preventing or treating thrombotic complications in COVID-19 patients.

## Abstract (web summary)

The serine protease factor Xa (FXa) is upregulated in COVID-19 patients and functions in the coagulation pathway. Here, Dong *et al* characterise the basis of its antiviral activity in the context of SARS-CoV-2 pandemic variants.

## Introduction

SARS-CoV-2 is the pathogen responsible for the global COVID-19 pandemic^1^. To date, approximately 680,000,000 cases and 6,800,000 deaths have been recorded, with a worldwide mortality rate of 2%^2^. Not surprisingly, the public health and economic consequences have been devastating. Although some strategies, such as FDA-approved vaccines and oral antiviral medications, have helped decrease morbidity and mortality among COVID patients, the pandemic rages on^3–5^.

Angiotensin-converting enzyme 2 (ACE2) is the host receptor for SARS-CoV-2^6,7^, which uses its spike (S) protein to bind to ACE2 and enter host cells. Cleavage of S protein to S1 and S2 subunits and then to S2′ is essential to initiate the membrane-fusion process^8^. For this purpose, the virus solicits the help of several host serine proteases (SPs)^9,10^. Furin cuts S protein at the PRRAR (R-R-A-R685↓) site into the S1 and S2 subunits at virus budding, while TMPRSS2 cleaves S protein at the S2′ site (P-S-K-R815↓) at viral entry; therefore, both cleavages are essential for SARS-CoV-2 infection^8,9,11,12^.

Another SP family member, activated coagulation factor X (FXa), binds to tissue factor to initiate the conversion of prothrombin to thrombin in the clotting cascade^13^. Direct FXa inhibitors (rivaroxaban, apixaban, edoxaban, and betrixaban), as well as an indirect inhibitor (fondaparinux), have been developed as clinical anticoagulants^14^, and several direct inhibitors are currently being evaluated for use in patients at high-risk for COVID-19^15^.

Here we show that FXa inhibits the entry of SARS-CoV-2 into cells. Mechanistically, FXa binds to and cleaves S protein, but with a different cleavage pattern than that produced by furin and TMPRSS2, and blocks S protein binding to ACE2. This inhibition of infectivity by FXa is found both in vitro and in vivo and is most pronounced with the ancestral WT SARS-CoV-2 virus but is diminished in variants such as B.1.1.7 and Delta that harbor the D614G mutation. Loss of endogenous FXa results in increased viral infection in vivo. Exogenous administration of FXa protects mice from lethal infection in a humanized hACE2 mouse model of COVID-19 when we use the WT SARS-CoV-2 but not the B.1.1.7 variant. The antiviral effect of FXa is attenuated by the direct FXa inhibitor rivaroxaban (RIVA) but not the indirect inhibitor fondaparinux (FONDA) both in vivo and in vitro.

## Results

### FXa inhibits infection of chimeric VSV-SARS-CoV-2 and authentic SARS-CoV-2

**Fig. 1:**
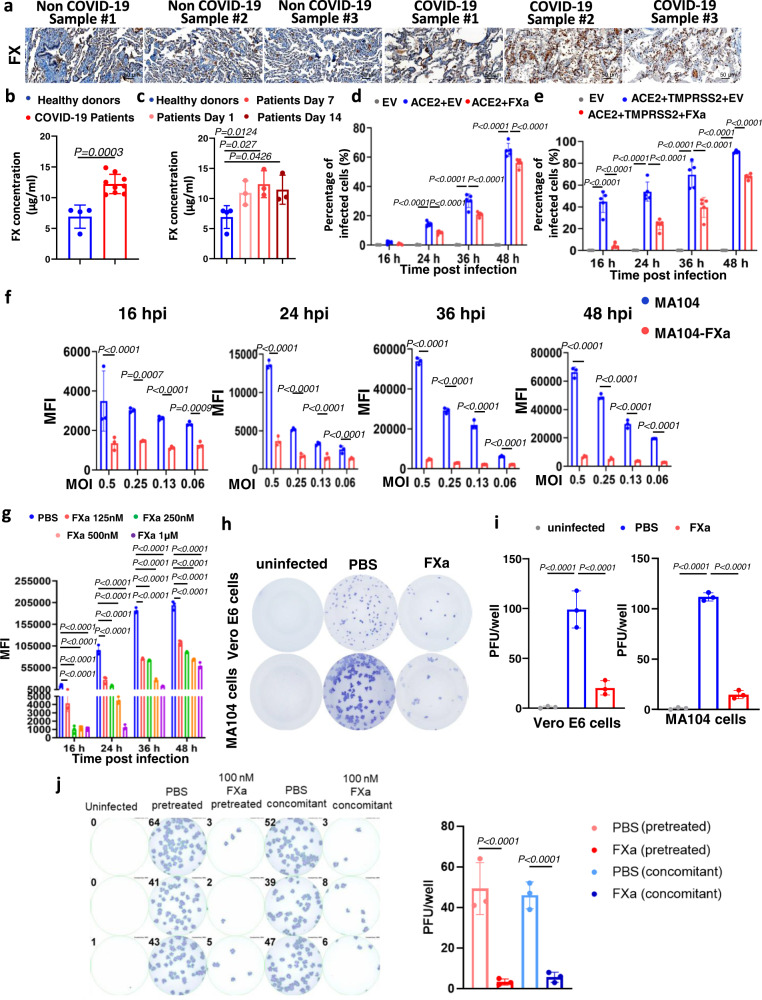
FXa inhibits wild-type SARS-CoV-2 infection by targeting viral particles. **a**, **b** FX protein levels in lungs (**a**) or plasma (**b**) of COVID-19 patients vs. non-COVID-19 donors, using IHC (**a**) and ELISA (**b**), respectively. The staining results shown in **a** are representative of at least two independent experiments with similar results. Scale bar, 50 μm. **b** *n* = 9 for COVID-19 patients and *n* = 4 for non-COVID-19 donors. **c** Post-diagnosis concentrations of FX in plasma of COVID-19 patients (*n* = 3) compared to non-COVID-19 donors (*n* = 3), as measured by ELISA. **d**, **e** HEK293T cells co-transfected with a plasmid encoding ACE2 and the other plasmid encoding FXa or an empty vector (EV) in the absence (**d**) or presence (**e**) of another plasmid encoding TMPRSS2 were infected by VSV-SARS-CoV-2. Infectivity of the cells was quantified by flow cytometry at 16, 24, 36, and 48 hpi (*n* = 4 or 5 biologically independent samples). **f** MA104 cells transduced with the plasmid encoding FXa (MA104-FXa) or an empty vector (MA104-EV) were infected with VSV-SARS-CoV-2. Infectivity of the cells was quantified by flow cytometry at 16, 24, 36, and 48 hpi (*n* = 3 biologically independent samples). **g** VSV-SARS-CoV-2 was pre-incubated with FXa at the indicated concentrations 1 hour before infection (*n* = 3 biologically independent samples). **h** MA104 and Vero E6 cells were infected with live wild-type SARS-CoV-2. At 24 hpi, infectivity was measured with an immuno-plaque assay. **i** Summary of data from **h** (*n* = 3 independent experiments for each cell line). **j** A549-ACE2 cells were infected with either authentic WT SARS-CoV-2 preincubated with 100 nM FXa for 1 hour, or the cells were treated with FXa at the time of viral infection. Infectivity was measured by immuno-plaque assays 24 hours post-infection. Representative infection and the summary data are presented at the left and right, respectively (*n* = 3 biologically independent samples). Data in **b**–**g** and **i**, **j** are presented as mean values ± standard deviation (SD) and statistical analyses were performed by two-sided Student’s *t* tests (**b**), one-way ANOVA models (**c**, **i**) and two-way ANOVA models (**d**–**g**, **j**). MFI data were log_2_ transformed before running the statistical models. Source data are provided as a Source Data file.

To identify changes in SPs during SARS-CoV-2 infection, we examined their expression in lung samples from COVID-19 patients using an immunohistochemical assay (IHC). Due to the lack of specific antibodies directly against FXa, we instead quantified FX, as ~100% of FX can be activated to FXa at injury sites when platelets are exposed to both collagen and thrombin^16^. Our IHC analysis indicated that thrombin was substantially higher in the lungs of COVID-19 patients compared to those without the disease (Supplementary Fig. 1a), as also previously reported^17,18^. We also found significantly increased levels of FX in the lungs of COVID-19 patients compared to non-COVID-19 donors; in contrast, we did not observe consistent upregulation of other tested SPs (Fig. 1a and Supplementary Fig. 1b). Moreover, compared to non-COVID-19 donors, COVID-19 patients’ FX levels were elevated in the liver and serum, which is the major source and carrier of FXa, respectively (Fig. 1b, c and Supplementary Fig. 1c). We analyzed the correlation between the expression of FXa and that of spike (S) protein in COVID-19 patients. Expression of FXa and S protein show a similar trend (Supplementary Fig. 1d).

To investigate the consequences of increased FXa during SARS-CoV-2 infection, we cloned FXa into the pCDH-mCherry vector and assessed its function, using a chimera of SARS-CoV-2 and the vesicular stomatitis virus, (VSV)-SARS-CoV-2^19^. HEK293T cells were co-transfected with ACE2 and FXa expression plasmids or control empty vector (EV). After 24 hours, cells were infected with VSV-SARS-CoV-2, and the percentages of infected cells (GFP-positive cells) were examined at the indicated time points using flow cytometry. Surprisingly, at the indicated hours post infection (hpi), the group transfected with the FXa expression plasmid had a significantly lower percentage of infected cells than the group transfected with EV (Fig. 1d). Thus, FXa efficiently blocked viral infection. However, SARS-CoV-2 infection depends not only on ACE2 but also on TMPRSS2^10^. When HEK293T cells were co-transfected with FXa, ACE2, and TMPRSS2 plasmids, FXa expression again blocked viral infection, showing a more pronounced effect during the early time points than without TMPRSS2 (Fig. 1e). To confirm our results, we generated an MA104 epithelial kidney cell line stably expressing FXa (MA104-FXa) (Supplementary Fig. 2a). The cells were infected with VSV-SARS-CoV-2 at different MOIs and the infectivity was determined at 16–48 hpi. Compared to the MA104-EV control cells, the MA104-FXa cells showed markedly less infection at each time point and at all MOIs (Fig. 1f and Supplementary Fig. 2b). Viral titers in supernatant from the infected MA104-FXa cells were significantly lower at 24 and 48 hpi than those from the MA104-EV cells, suggesting that overexpression of FXa also impaired viral production (Supplementary Fig. 2c, d). We also compared the role of FXa with that of other SPs in preventing viral infection of parental MA104 cells. Unlike pre-treatment with furin, TMPRSS2, or trypsin, all of which increased VSV-SARS-CoV-2 infection at low doses, pre-treatment with FXa reduced viral infection regardless of the dose (Supplementary Fig. 3a–c). Consistent with this, FXa significantly decreased the viral titer in supernatant from MA104 cells infected with VSV-SARS-CoV-2, while furin, TMPRSS2, or trypsin increased the viral titer compared to vehicle control (PBS) (Supplementary Fig. 3d). We also explored the role of FXa on viral infection when the SARS-CoV-2 was bound to cell receptors. For this purpose, VSV-SARS-CoV-2 virus was added to MA104 cells. One hour later, the media with free VSV-SARS-CoV-2 virus was removed, and cells with bound virus were washed twice. The cells with the bound virus were then treated with FXa or PBS (control). Our data showed that compared to PBS, FXa treatment still inhibited viral infection when SARS-CoV-2 was already bound to cell receptors (Supplementary Fig. 3e).

Furthermore, to determine if FXa blocks viral infection by targeting SARS-CoV-2 or host cells, we first constructed an FXa-Fc fusion protein expression plasmid and purified the protein from Chinese hamster ovary (CHO) cells. We next co-incubated VSV-SARS-CoV-2 with or without FXa-Fc fusion protein in vitro for 1 hour before adding the mixture into MA104 cells. By determining the infection rate at the indicated time points, we found that preincubating VSV-SARS-CoV-2 with the FXa-Fc fusion protein significantly inhibited viral infection in a dose-dependent manner (from 62.5 nM to 1 μM) (Fig. 1g and Supplementary Fig. 4a). This suggests that FXa blocked viral infection by targeting SARS-CoV-2. Of note, inactivated FXa had no effect on the viral infection (Supplementary Fig. 4a). To confirm the effects of FXa on viral infectivity, we infected MA104 cells with VSV-SARS-CoV-2 at a very low MOI (0.001) in the presence or absence of purified FXa protein at the indicated concentrations. We found that the viral infectivity decreased in a dose-dependent manner as the concentration of FXa increased (Supplementary Fig. 4b), suggesting that FXa plays an essential role in inhibiting viral infectivity.

To assess whether endogenous or natural FXa in human peripheral blood exhibits antiviral activity against the SARS-CoV-2 coronavirus, we first converted FX into its active form, FXa, in plasma from healthy donors. Next, MA104 cells were infected with VSV-SARS-CoV-2 chimeric virus that had been pre-treated with human plasma unconverted or converted from FX to FXa. The converted plasma significantly decreased infection with the chimeric VSV-SARS-CoV-2 virus (Supplementary Fig. 4c). We also performed the infection experiment with the tissue factor (TF)-FVIIa-FXa complex and used the VSV-SARS-CoV-2 chimera. The TF-FVIIa-FXa complex inhibited infection by the chimeric virus compared to PBS, FVIIa, and TF controls, indicating that FXa in a natural complex can also reduce viral infection (Supplementary Fig. 4d).

To determine whether FXa also inhibits viral infection by interacting with host cells, we preincubated different concentrations of FXa-Fc fusion protein with MA104 cells for 1 hour, washed out media, and then infected the cells with VSV-SARS-CoV-2. We found that FXa-Fc fusion protein pre-treatment with the MA104 cells did not significantly affect viral infection (Supplementary Fig. 5a). We next used live SARS-CoV-2 to infect Vero E6 and MA104 cells followed by quantitative assessment of viral load using an immuno-plaque assay. We found that pre-incubation of 100 nM FXa with authentic SARS-CoV-2 prior to infection significantly reduced viral infection in both Vero E6 and MA104 cells compared to the buffer control, consistent with the above data from the chimeric VSV-SARS-CoV-2 virus (Fig. 1h, i). Given that lung cells are more physiologically relevant to COVID-19 than Vero E6 and MA104 cells, we infected the A549 lung cell line expressing ACE2 (A549-ACE2) with authentic SARS-CoV-2 while in the presence or absence of FXa. We observed that concomitant treatment of FXa and authentic SARS-CoV-2 in A549-ACE2 cells gave results that were similar to those obtained when authentic SARS-CoV-2 was pre-treated with FXa before attempting to infect A549-ACE2 cells (Fig. 1j). Collectively, our results show that FXa, an SP that is upregulated following SARS-CoV-2 infection in host cells, inhibits SARS-CoV-2 infection and thus possesses an antiviral activity, in distinct contrast to other SPs such as furin, trypsin, and TMPRSS2.

### FXa suppresses viral entry by binding to and cleaving the SARS-CoV-2 S protein

**Fig. 2:**
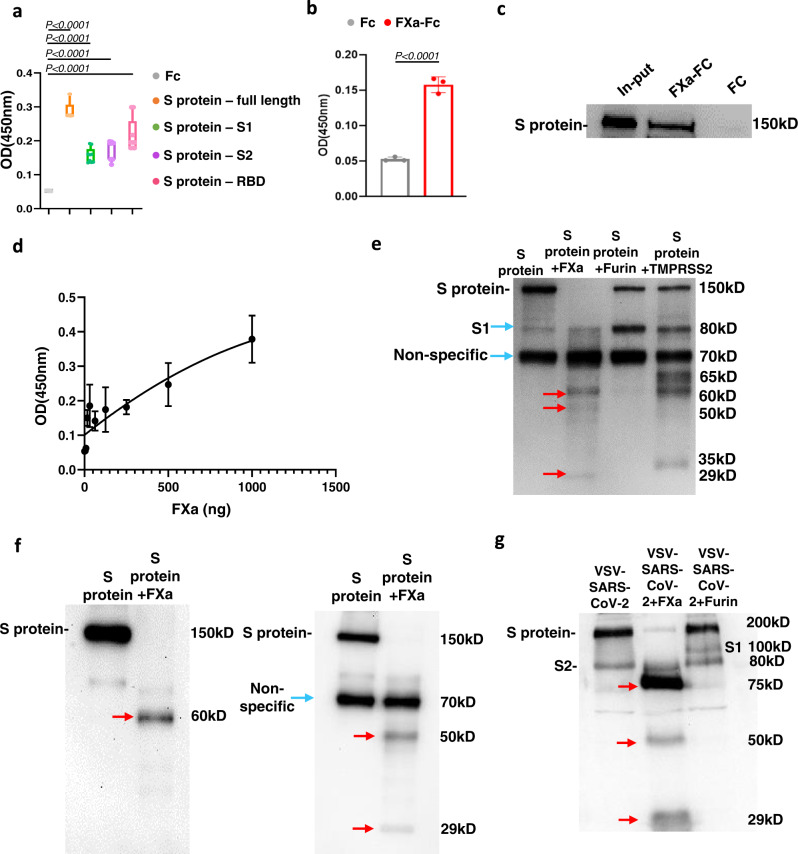
FXa suppresses viral entry by binding to and cleaving the SARS-CoV-2 S protein. **a** The binding affinity of FXa with full-length wild-type S protein, subunit S1, subunit S2, or RBD was quantified by ELISA (*n* = 9 biologically independent samples). Bounds of box is from 25% percentile to 75% percentile, horizontal bar indicates median, and whiskers indicate data ranges. **b** The binding affinity of FXa to VSV-SARS-CoV-2 viral particles was quantified by ELISA (*n* = 3 biologically independent samples). **c** The interaction between FXa protein and full-length S protein was examined with a pull-down assay. **d** The binding affinity at indicated concentrations of FXa was measured by ELISA (*n* = 3 biologically independent samples). **e** The cleavage of S protein by furin, TMPRSS2, and FXa after 3-hour incubation was analyzed by immunoblotting using an anti-S protein antibody (40591-T62, Sino Biological). **f** S protein was cleaved by FXa, followed by immunoblotting with an anti-RBD antibody (MAB10540-100, R&D) (left) and an anti-S2 antibody (MA5-35946, Invitrogen) (right). **g** The cleavage of VSV-SARS-CoV-2 by FXa or furin was analyzed by immunoblotting using an anti-S protein antibody (40591-T62, Sino Biological). The larger size of S protein from VSV-SARS-CoV-2 (~200 kD) compared to recombinant S protein (~150 kD) may be due to the glycosylation of the former. All data are representative of at least three independent experiments. **a**, **b**, **d**, data are presented as mean values ± SD. Statistical analyses were performed by two-sided Student’s *t* test (**b**) or one-way ANOVA models (**a**). Source data are provided as a Source Data file.

To study the mechanism(s) of the antiviral activity of FXa, we compared the binding between FXa and various subunits of the SARS-CoV-2 S protein. Compared to control Fc protein, FXa had the strongest binding affinity toward the full-length S protein and, to a lesser extent, to subunit S1, subunit S2, and the receptor binding domain (RBD) (Fig. 2a). ELISA confirmed the strong binding affinity between VSV-SARS-CoV-2 viral particles and FXa (Fig. 2b), and a pull-down assay showed that FXa but not Fc control protein co-precipitated with S protein (Fig. 2c). The binding affinity of FXa to S protein was in the nanogram range and was dose-dependent manner (Fig. 2d). We also measured the binding affinity between active or heat-inactivated FXa with S protein or with VSV-SARS-CoV-2. Our data show there is no difference in binding when comparing active FXa to heat-inactivated FXa (Supplementary Fig. 5b, c). These results suggest that both active and inactive FXa binds to S protein.

As the virus enters host cells, its proteins undergo important conformational changes as host SPs cleave S protein. To determine whether FXa can cleave S protein, we incubated full-length S protein with FXa for three hours and then used immunoblotting. Furin and TMPRSS2 served as positive controls, as they are known to induce functional conformational changes in S protein. We found that full-length S protein was cut into three fragments by FXa with the sizes of ~60 kD, 50 kD, and 29 kD (Fig. 2e). The 60 kD fragment can be detected by an anti-spike RBD antibody (Fig. 2f, left), while both 50 kD and 29 kD fragments were detected by an anti-spike S2 subunit antibody (Fig. 2f, right). This cleavage pattern was in contrast to that of furin and TMPRSS2, which both cut full-length S protein into the subunit S1 (Fig. 2e). As noted above, cleavage by FXa did not produce the S1 subunit. Of note, a non-specific band of S protein produced from baculovirus-insect cells is present without treatment and liquid chromatography-mass spectrometry (LC-MS)/MS analysis indicated that it is a part of S protein consisting of non-continuous fragments (Supplementary Data File 1). Thus, that band is not a cleaved protein product and is labeled as “Non-specific”. We next performed a cleavage assay of the native S protein on virus particles by FXa. For this purpose, VSV-SARS-CoV-2 chimeric viral particles were incubated with FXa. Furin was included as control, which assumably cleaved the VSV-SARS-CoV-2 virus into S1 and S2 fragments. The immunoblotting assay data showed that VSV-SARS-CoV-2 virus was cleaved into three fragments by FXa with the sizes of approximately 75 kD, 50 kD, and 29 kD (Fig. 2g). These fragments resembled those detected in the cleavage assay using recombinant S protein (Fig. 2e). That cleavage pattern is consistent with the in silico prediction of two FXa cleavage sites on S protein: Gly-Arg (R567) and Ile-(Asp/Glu)-Gly-Arg (R1000) (Supplementary Fig. 5d). Of note, the appearance of a 75 kD fragment, instead of a 60 kD one, in the cleavage assay of VSV-SARS-CoV-2 virus could have resulted from glycosylation of S at its N-terminal, as S proteins on native viral particles should be glycosylated trimers^20^. For the furin control, as expected, the cleavage of viral particles by it generated an S1 band (Fig. 2g).

We performed the shedding experiment with S protein to clarify the mechanism underlying FXa-mediated inhibition of SARS-CoV-2 entry. For this purpose, A549 cells were transduced with a pCDH lentiviral vector expressing S protein, which is referred to as A549-S cells. GFP was co-expressed with S protein for FACS-sorting to purify transduced cells. 25 nM or 1 μM FXa was used to treat the cells in PBS for 12 hours. After FXa treatment, the supernatants from each group were collected to measure S protein by ELISA with anti-RBD antibody and immunoblotting assay with anti-S antibody. The expression of S protein on the surface of A549-S cells was detected by FACS. GFP was detected as a control as it cannot be cleaved by FXa. As expected, our data showed that, compared to the untreated control group, FXa treatment decreased S protein on the A549-S cell surface while increasing S protein concentration in supernatants, both in a dose-dependent manner (Supplementary Fig. 5e, f). The immunoblotting assay result of the supernatant showed that the 60 kD and 50 kD fragments existed but without the 29 kD fragment (Supplementary Fig. 5g). The reason may be that R1000 cleavage site is near the transmembrane domain of S protein^21^, which may result in retaining the 29 kD fragment on the cell surface.

### FXa cleavage reduces the binding between S protein and ACE2

**Fig. 3:**
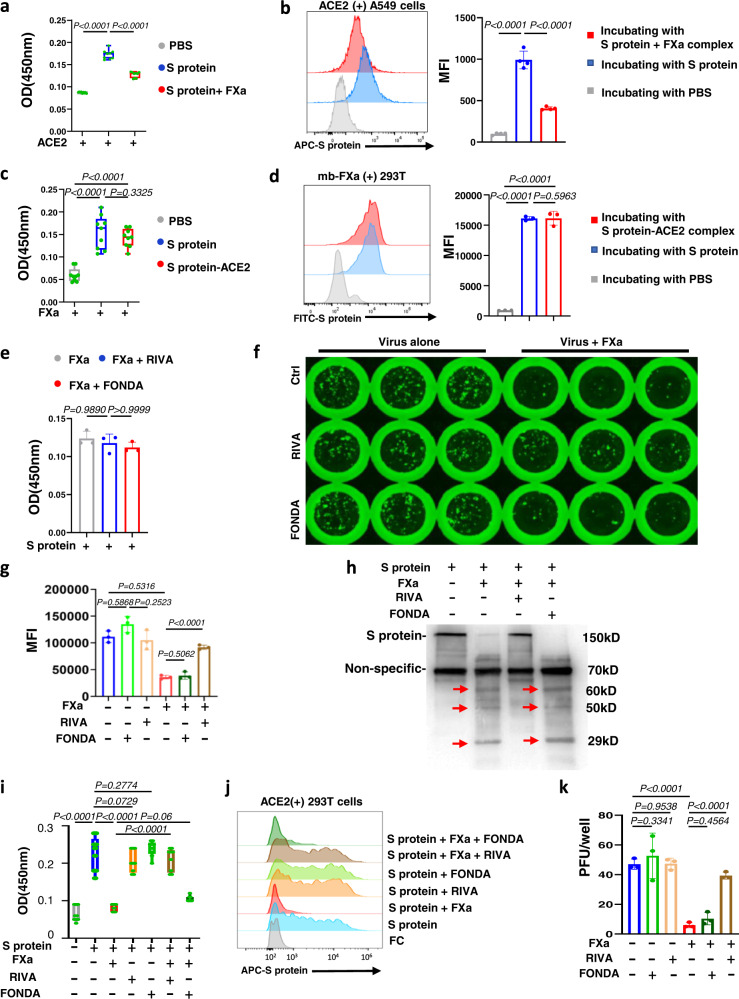
FXa cleavage reduces the binding between S protein and ACE2. **a** The binding between ACE2 with S protein or FXa-pre-treated S protein was measured by ELISA (*n* = 9 biologically independent samples). **b** The binding between S protein or FXa-pre-treated S protein and ACE2 expressed on A549 human lung cancer cells was measured by flow cytometry (left, representative flow cytometry histogram; right, summary data) (*n* = 4 independent experiments). **c** The binding of FXa with S protein, S protein–ACE2 complex, or PBS control was measured by ELISA (*n* = 9 biologically independent samples). **d** The binding of membrane-bound (mb) FXa with S protein or the binding of mb FXa with S protein–ACE2 complex on 293 T cells was measured by flow cytometry (left, representative flow cytometry histogram; right, summary data) (*n* = 3 independent experiments). PBS served as control for S protein and the S protein–ACE2 complex. **e** The effect of RIVA or FONDA on the binding of FXa with S protein was measured by ELISA (*n* = 3 independent experiments). **f**, **g** Infectivity of VSV-SARS-CoV-2 in MA104 cells that were pre-treated or not treated with FXa in the presence or absence of RIVA or FONDA was examined with fluorescent microscopy (**f**) and flow cytometry (**g**, *n* = 3 biologically independent samples). **h** S protein cleavage by FXa was examined by immunoblotting 3 hours after their incubation in the presence or absence of RIVA or FONDA, using an anti-S protein antibody (40591-T62, Sino Biological). **i**, **j** FXa pre-treated or not treated with RIVA or FONDA was incubated with S protein; then the binding capability of those S proteins with ACE2 coated on a plate (**i**, *n* = 9 biologically independent samples) or ACE2 expressed on 293 T cells (**j**) was assessed. **k** Infectivity of live SARS-CoV-2 in A549-ACE2 cells pre-treated or not treated with FXa in the presence or absence of RIVA or FONDA was examined using an immuno-plaque assay (*n* = 3 independent experiments). **a**, **c**, **i**, the top and the bottom bound of box are 75th percentile and 25th percentile, respectively; horizontal bar indicates median; and whiskers indicate data ranges. Experiments in **h** and **j** are representative of three independent experiments with similar data. For all panels, data are presented as mean values ± SD, and statistical analyses were performed by one-way ANOVA models. MFI data were log_2_ transformed before running the statistical models. Source data are provided as a Source Data file.

Given that FXa could cut S protein into different fragments, we used an ELISA to determine whether S protein cleavage by FXa affected the binding of S protein to ACE2. The ELISA data indicated that when S protein was pre-treated with FXa it had a lower binding affinity to ACE2 compared to untreated S protein (Fig. 3a). Flow cytometry confirmed this result, demonstrating that S protein pre-treated with FXa could not efficiently bind to ACE2-expressing A549 or HEK293T cells (Fig. 3b and Supplementary Fig. 6a). We also showed that FXa could still bind to S protein after the latter had already bound to ACE2 (Fig. 3c d) and that it could still cleave S protein (Supplementary Fig. 6b). Overall, our results imply that FXa could be an efficient inhibitor of viral entry because it cleaves S protein in a manner that prevents the virus from entering cells.

### The FXa direct inhibitor rivaroxaban but not the indirect inhibitor fondaparinux blocks FXa-induced antiviral activity in vitro

COVID-19 patients with an increased risk of thrombosis are treated with direct FXa inhibitors (e.g., rivaroxaban) or indirect inhibitors (e.g., fondaparinux)^15,22^. We, therefore, asked if rivaroxaban (RIVA) or fondaparinux (FONDA) affects the antiviral activity of FXa. Neither RIVA nor FONDA alone substantially blocked the binding of FXa to S protein (Fig. 3e) and neither drug alone had any effect on VSV-SARS-CoV-2 infectivity (Fig. 3f left). However, the direct FXa inhibitor RIVA blocked FXa-induced antiviral activity, whereas the indirect FXa inhibitor FONDA did not (Fig. 3f right and g). We also measured viral titers in supernatants collected from this inhibitor experiment, again confirming that RIVA but not FONDA significantly reduced FXa-induced antiviral activity (Supplementary Fig. 7a). Furthermore, a cleavage assay showed that the direct FXa inhibitor RIVA but not the indirect inhibitor FONDA inhibited cleavage of S protein by FXa (Fig. 3h). Consistent with this observation, pre-treating a mixture of S protein and FXa with RIVA or FONDA and then incubating the mixture with ACE2 showed that RIVA—but again not FONDA—significantly diminished FXa’s ability to inhibit the binding of S protein to ACE2, as demonstrated by ELISA, presumably by inhibiting cleavage of S protein by RIVA but not by FONDA (Fig. 3i). The ELISA results in Fig. 3i were validated by flow cytometric analysis (Fig. 3j and Supplementary Fig. 7b). Our data imply that the direct rather than indirect inhibitor of FXa could diminish FXa-mediated blockade of viral entry via inhibiting the cleavage of S protein by FXa, allowing intact S protein to efficiently bind to ACE2. An infectivity assay using live SARS-CoV-2 and A549-ACE2 cells gave similar results (Fig. 3k).

### Exogenous FXa inhibits while FXa knockout promotes SARS-CoV-2 infection in the K18-hACE2 mouse model

**Fig. 4:**
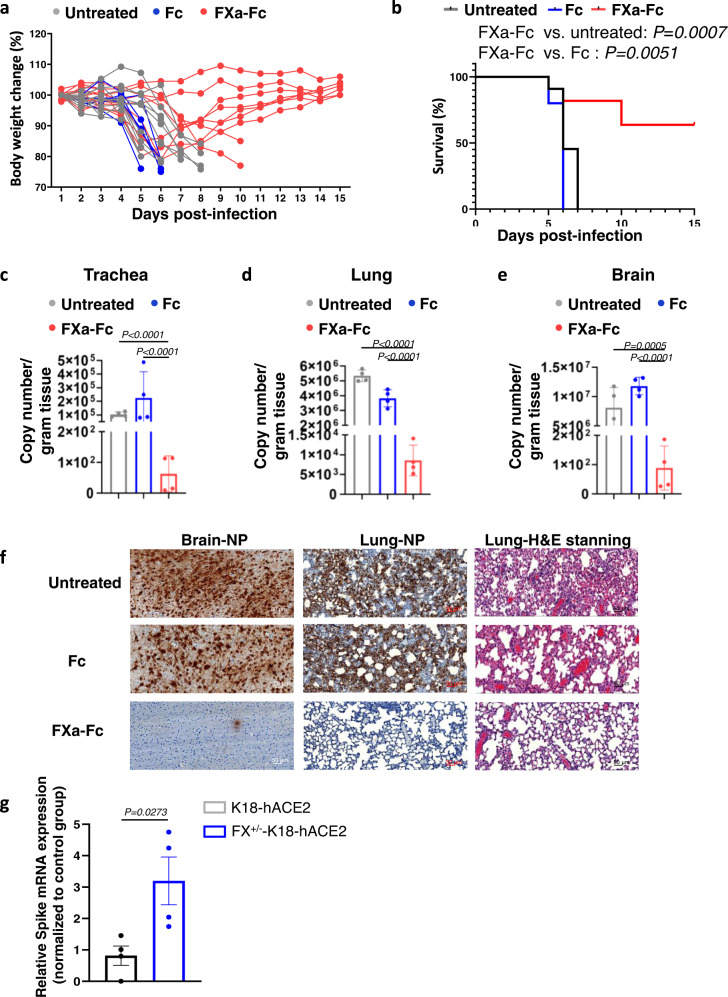
The in vivo effect of FXa protein and heterozygous knockout on WT SARS-CoV-2 infection in a K18-hACE2 mouse model of COVID-19. **a**, **b** Body weight (**a**) and survival (**b**) of mice infected with 5 × 10^3^ PFU SARS-CoV-2 WT strain and treated with or without 200 µg FXa-Fc fusion protein. Fc-protein alone was used as control. *N* = 11 mice for PBS and FXa-Fc group. *N* = 5 mice for Fc group. **c**–**e** Relative viral copy numbers in the tracheas (**c**), lungs (**d**), and brains (**e**) of mice treated with or without FXa-Fc fusion protein were assessed by qPCR. Fc protein alone served as control. *N* = 4 biologically independent mice per group. **f** SARS-CoV-2 was detected in the brain and lung of mice treated with FXa-Fc or Fc-protein by using IHC staining with an antibody against SARS-CoV-2 nucleocapsid protein (NP). Pathological analysis by H&E staining of lungs of untreated mice and mice treated with FXa-Fc or Fc-protein alone. The experiment was repeated with four mice per group with similar results. Scale bar, 50 μm. The untreated and Fc-protein-treated mice were sacrificed on day 5 post infection given their declining health; as the FXa-Fc group survived for more than 2 weeks, their tissues were collected on day 15 post infection (**e**, **f**). **g** S protein mRNA levels in lungs of FXa^+/−^-K18-hACE2 mice and WT K18-hACE2 mice (littermate control) were assessed by qPCR. *N* = 4 biologically independent mice per group. Data are presented as mean values ± SD and statistical analyses were performed by one-way ANOVA models (**c**–**e**), two-sided Student’s *t* test (**g**), or log-rank test (**b**). Source data are provided as a Source Data file.

To evaluate the potential effect of FXa in vivo, we used humanized K18-hACE2 mice as an infection model of SARS-CoV-2^23,24^. We inoculated those mice with SARS-CoV-2 and then treated them intranasally with FXa-Fc protein or two controls, saline and the Fc protein (200 µg/mouse for each protein). The body weight of the mice was monitored. Most of the mice in the untreated and Fc-treated groups exhibited a dramatic decrease in their body weight 5 days post infection, and they were euthanized at that time or shortly thereafter. In contrast, body weight of the majority of mice treated with FXa-Fc either did not change or started to regain after an initial drop (Fig. 4a). The FXa-Fc-treated group also lived significantly longer than the two control groups, with no difference between the control groups (Fig. 4b). To measure copy numbers in trachea, lung, and brain tissue, we isolated RNA and used quantitative real-time PCR. The copy number in the FXa-Fc-treated group was ~1000-fold lower than in the two control groups, indicating that FXa-Fc had significantly restricted SARS-CoV-2 infection in vivo (Fig. 4c–e). Consistent with this, IHC showed that expression of the SARS-CoV-2 viral nucleocapsid protein (NP) was also markedly lower in brain and lung tissues in the FXa-Fc-treated group compared to the untreated and Fc-treated groups (Fig. 4f). The histological study showed that FXa-Fc-treated mice had more intact lung structure and less pathological damage than the two control groups (Fig. 4f). We also treated mice with 100-fold less FXa per mouse, i.e., 2 µg per mouse, using vehicle as a negative control. 2 µg FXa treatment still showed a protection ratio 33.3% at day 15 post treatment while all mice died by day 7 in the vehicle control group (Supplementary Fig. 8).

The effect of low-dose FXa in protecting mice from SARS-CoV-2 infection, as described above, prompted us to investigate the potential endogenous role of FXa on SARS-CoV-2 infection using a knockout approach. For this purpose, we generated FX knockout mice by CRISPR/Cas9 gene-editing technology. We crossed the mice with K18-hACE2 mice and obtained the FX (heterozygote)-K18-hACE2 strain. FX was knockdown successfully in the heterozygotes, confirmed by qPCR and immunoblotting assay (Supplementary Fig. 9a, b), while FX homozygote knockouts are embryonic lethal. To clarify endogenous FXa function, we inoculated FX (heterozygote)-K18-hACE2 mice and K18-hACE2 mice (littermate control) with SARS-CoV-2, followed by measuring S protein mRNA levels of lung tissues. The results showed that the S protein mRNA level in the lungs of FX (heterozygote)-K18-hACE2 were significantly increased compared to the K18-hACE2 control group, suggesting that the endogenous FXa has anti-SARS-CoV-2 infectivity properties (Fig. 4g). These data, together with the infection inhibition of converted FXa from human plasma (Supplementary Fig. 4c), substantiate the endogenous anti-SARS-CoV-2 role of FXa.

### The FXa direct inhibitor rivaroxaban but not the indirect fondaparinux abrogates FXa-mediated protection of K18-hACE2 mice from WT SARS-CoV-2 infection

**Fig. 5:**
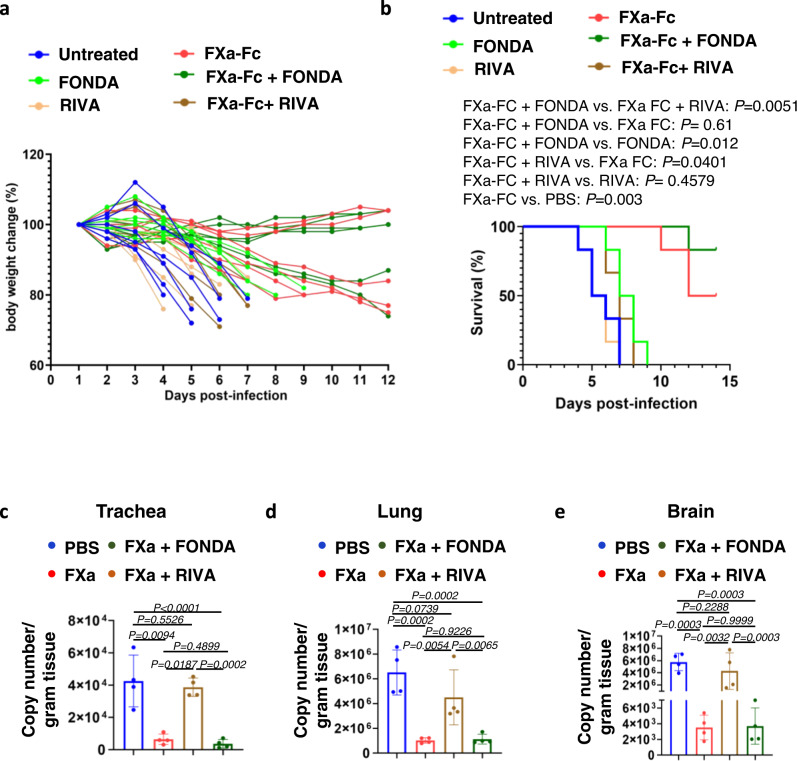
Effect of the direct FXa inhibitor RIVA and the indirect inhibitor FONDA on FXa-mediated protection of K18-hACE2 mice from WT SARS-CoV-2 infection. **a**, **b** Body weight (**a**) and survival (**b**) of mice infected with 5 × 10^3^ PFU of SARS-CoV-2 (WA1) and treated with or without FXa-Fc in the presence or absence of RIVA or FONDA. *N* = 6 mice for each group. **c**–**e** Relative viral copy numbers in the trachea (**c**), lung (**d**), and brain (**e**) of mice treated with or without FXa-Fc in the presence or absence of RIVA or FONDA were assessed by qPCR. All the mice were sacrificed on day 5 post infection. *N* = 4 mice for each group. **c**–**e** Data are presented as mean values ±SD. Statistical analyses were performed by one-way ANOVA models (**c**–**e**) or log-rank test (**b**). Copy number (**c**–**e**) was log_2_ transformed before running statistical models. Source data are provided as a Source Data file.

To evaluate the direct FXa inhibitor RIVA and the indirect FXa inhibitor FONDA in vivo, we administered them intranasally into SARS-CoV-2-infected mice treated with FXa. Consistent with the in vitro data, we found that the direct FXa inhibitor RIVA significantly blocked the antiviral and survival advantage afforded by intranasal administration of FXa-Fc. In contrast, the indirect FXa inhibitor FONDA had no significant effect on the antiviral and survival advantage afforded by intranasal administration of FXa-Fc alone (Fig. 5a–e). The NP IHC data and histological study also showed that RIVA abolished FXa’s antiviral infection function while FONDA had no such effect (Supplementary Fig. 10). In the absence of a prospective, double-blind randomized clinical trial comparing the direct and indirect inhibitors of FXa in COVID-19 patients, these in vivo results with live SARS-CoV-2 provide preclinical support for using an indirect FXa inhibitor such as FONDA as an anticoagulant when attempting to prevent or treat thrombotic complications of COVID-19. They also suggest that a direct FXa inhibitor, such as the anticoagulant RIVA, should be avoided under such clinical circumstances as it would likely negate any beneficial effects of FXa.

### FXa is less effective in blocking infection of the SARS-CoV-2 B.1.1.7 variant compared to WT strain in vitro and in vivo

**Fig. 6:**
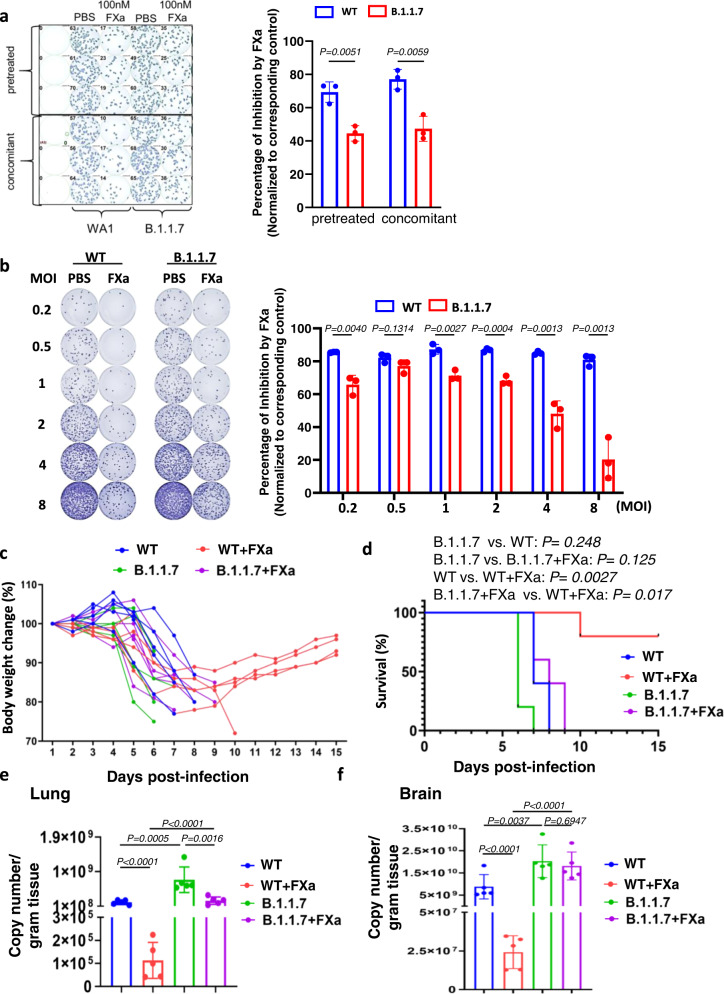
FXa is less effective in blocking infection of the SARS-CoV-2 B.1.1.7 variant in vitro and in vivo. **a** A549-ACE2 cells were preincubated or not preincubated with 100 nM FXa for 1 h, and then infected with either live SARS-CoV-2 WA1 or the SARS-CoV-2 B.1.1.7 variant. Infectivity was measured with an immuno-plaque assay 24 hours post infection and the infection inhibition ratio induced by FXa was summarized (right panel). *N* = 3 biologically independent samples. **b** Vero E6 cells were pre-treated with FXa and then infected with live SARS-CoV-2 WA1 or the SARS-CoV-2 B.1.1.7 variant at various MOIs. At 24 hpi, infectivity was measured with an immuno-plaque assay (left panel), and the infection inhibition ratio induced by FXa at different MOIs was summarized (right panel). *N* = 3 biologically independent samples. **c**, **d** Body weight (**c**) and survival (**d**) of mice infected with 5 × 10^3^ PFU wild-type SARS-CoV-2 or B.1.1.7 variant and treated with or without FXa-Fc fusion protein. **e**, **f** Viral load in the lung (**e**) and brain (**f**) of mice treated with or without FXa-Fc fusion protein, was assessed by qPCR. All the mice were sacrificed at day 5 post infection. *N* = 5 biologically independent mice (**c**–**f**). Data in **a**, **b**, **e**, **f** are presented as mean values ± SD. Statistical analyses were performed by two-way ANOVA models (**a**, **b**), one-way ANOVA models (**e**, **f**), or log-rank test (**d**). Copy number (**e**, **f**) was log_2_ transformed before running statistical models. Source data are provided as a Source Data file.

The B.1.1.7 SARS-CoV-2 variant, Alpha, which emerged in the United Kingdom in September 2020, has many mutations associated with increased transmissibility and higher risk of death^25,26^. To evaluate FXa’s effectiveness against that variant, we infected the A549-ACE2 cells with the original emergent SARS-CoV-2 (WA1; WT) or the B.1.1.7 variant that had been pre-treated or concomitantly treated with FXa. The immuno-plaque results showed that FXa was less efficient in inhibiting infection by the B.1.17 variant compared to WT virus (Fig. 6a). Furthermore, the results were confirmed by viral infection at various MOIs, which showed that FXa could still block WT infection even at a very high MOI (MOI = 8) but had little effect on B.1.1.7 infection blockade at the same MOI (Fig. 6b).

We also tested the effects of the direct (RIVA) and indirect (FONDA) FXa inhibitors on the B.1.1.7 SARS-CoV-2 variant using the WT strain as control. We found that RIVA efficiently blocked the antiviral effect of FXa in a dose-dependent manner, starting as low as 0.05 µg/ml dose, while FONDA did not even at the high dose of 50 µg/ml, against the B.1.1.7 variant infection in both Vero E6 and MA104 cells (Supplementary Fig. 11a and 12a). These data were validated in similar experiments with dose concentration gradients of FXa in both Vero E6 and MA104 host cells (Supplementary Fig. 11b and 12b).

Next, we compared the antiviral effect of FXa against the WT and B.1.1.7 SARS-CoV-2 variant in vivo. Consistent with the in vitro data, we found that the antiviral and survival advantage afforded by FXa-Fc was abolished or significantly decreased in the B.1.1.7 variant-infected group compared to the WT-infected group (Fig. 6c–f). Thus, our data showed that the B.1.1.7 variant, with its mutated spike protein, was less efficient in FXa-mediated infectivity inhibition in vitro and in vivo compared to the WT strain.

### FXa is less effective in blocking infection of the SARS-CoV-2 variants with the D614G mutation compared to WT strain

**Fig. 7:**
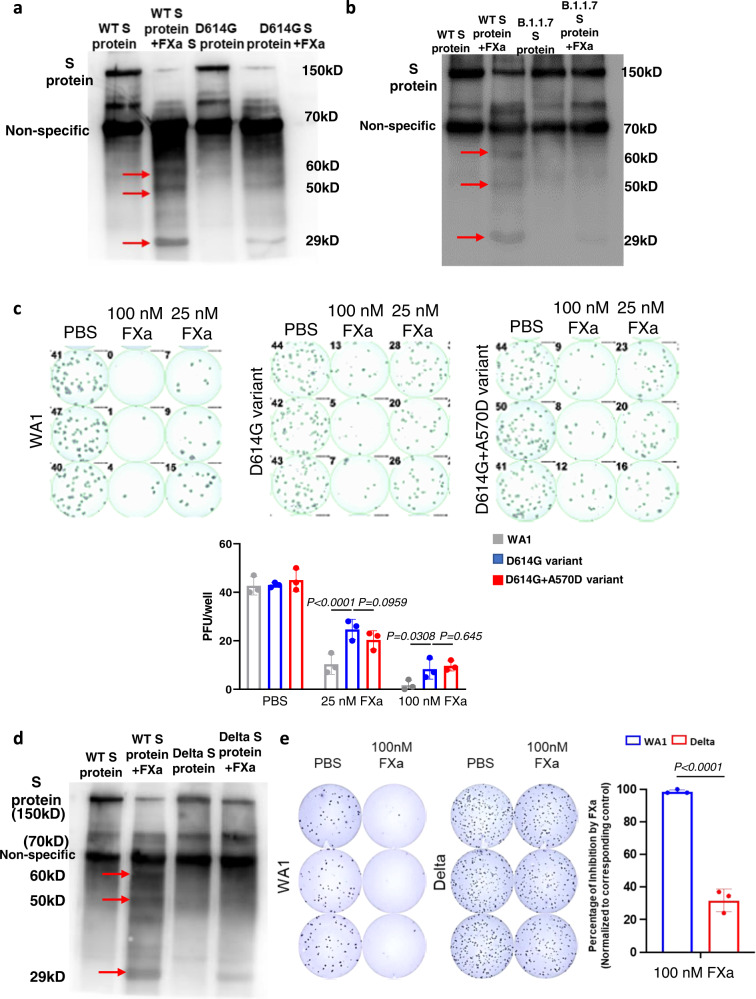
FXa is less effective in blocking infection of the SARS-CoV-2 variants with the D614G mutation. **a** Cleavage of WT WA1 S protein or D614G S protein by FXa after 1-hour incubation was analyzed by immunoblotting using an anti-S protein antibody (40591-T62, Sino Biological). **b** Cleavage of WT WA1 S protein or B.1.1.7 S protein by FXa after 1-hour incubation was analyzed by immunoblotting using the same antibody in **a**. **c** Live SARS-CoV-2 WT WA1, the D614G variant, and the D614G variant engineered with an A570D mutation (D614G + A570D) were treated with 100 nM or 25 nM FXa in A549-ACE2 cells. Infectivity was measured with an immuno-plaque assay 24 hours post infection. **d** Cleavage of WT WA1 S protein or Delta S protein by FXa after 1-hour incubation was analyzed by immunoblotting using the same antibody in **a**. **e** Live SARS-CoV-2 WT WA1 and the Delta variant were treated with 100 nM FXa in A549-ACE2 cells. Infectivity was measured with an immuno-plaque assay 24 hours post infection) (left) and then summarized in graphical form (right). *N* = 3 biologically independent samples **c**, **e**. Immunoblotting data are representative of three independent experiments (**a**, **b**, **d**). Data in **c** and **e** are presented as mean values ± SD and statistical analyses were performed by two-way ANOVA models (**c**) or two-sided Student’s *t* test (**e**). Source data are provided as a Source Data file.

To explore the mechanism of this difference of FXa targeting different variants, we compared the binding affinity between FXa and WT S protein with that between FXa and B.1.1.7 S protein. Both ELISA and flow cytometry results showed that FXa had a significantly lower binding affinity with B.1.1.7 S protein compared with WT S protein (Supplementary Fig. 13a, b). As B.1.1.7 has several mutations in its S protein, we determined which one was most important for resisting binding and cleavage by FXa. We focused on the aspartic acid–614 to glycine (D614G) substitution in S protein, as it had been linked to enhanced SARS-CoV-2 infection^27,28^. We determined whether FXa had a similar functional interaction with the D614G S protein as with the WT S protein. FXa could still bind to and cleave the D614G S protein (Supplementary Fig. 13c–e), and the binding affinity between ACE2 and D614G S protein decreased if the D614G S protein was pre-treated with FXa (Supplementary Fig. 13f, g). However, FXa cleaved the D614G S protein less efficiently than it cleaved the WA1 S protein after one-hour incubation (Fig. 7a). This implied that the D614G variant might be resistant to FXa-mediated antiviral activity. When we compared cleavage of the WA1 and B.1.1.7 S proteins by FXa, we found less efficient cleavage of B.1.1.7 S protein by FXa compared to WA1 S protein after one-hour incubation (Fig. 7b).

As both D614G and A570D in the B.1.1.7 variant are close to FXa’s predicted cleavage site 567, we next tested whether either or both mutations are responsible for the reduction in FXa-mediated antiviral activity. For this purpose, a custom variant carrying both D614G and A570D was used, referred to as “D614G + A570D”^29,30^ or D614G alone in the WA1 background. We infected Vero E6 cells with the original emergent WA1 SARS-CoV-2 (WT), the D614G variant, or the custom-made variant D614G + A570D. The immuno-plaque results revealed that FXa showed significantly less antiviral activity against the D614G variant compared to the WT strain. However, there was no difference between the D614G variant and the D614G + A570D custom-made variant after FXa treatment, indicating that the D614G mutation was the important mutation for significantly reducing FXa’s antiviral activity (Fig. 7c). We also performed cleavage assays with S protein of the Delta and Omicron variants, both of which contain the D614G mutation relative to WA1. Similarly, the cleavage of S protein of Delta and Omicron variants by FXa was less efficient than that of the WA1 S protein after one-hour incubation (Fig. 7d and Supplementary Fig. 13h). We also compared the viral infection of the WA1 and the Delta SARS-CoV-2 variants in the presence or absence of FXa. As we expected, Delta variant, which contains the D614G mutation, showed much less efficiency for FXa-depended inhibition of SARS-CoV-2 infection when compared to WA1 strain (Fig. 7e).

Collectively, our experimental data demonstrate that variant strains carrying the D614G mutation in their S protein (all dominant pandemic variants to date) are relatively resistant to the FXa antiviral effect compared to the WT strain. This observation may in part explain the emergence and higher transmission rates of variants containing the D614G mutation^27,31^.

## Discussion

Here, we identify a mechanism of human host antiviral defense involving the human SP FXa, which, at the time of SARS-CoV-2 infection, binds to and cleaves the SARS-CoV-2 S protein, blocking viral entry into host cells. In contrast to other SPs, the precursor to FX was found to be increased in COVID-19 patient tissues and serum compared to normal donors. In an in vivo experimental mouse model, a decrease in endogenous FXa increased mRNA levels of S protein following infection with SARS-CoV-2. Exogenous administration of FXa reduced viral copy numbers and protected a humanized hACE2 mouse model of COVID-19 from lethal infection, an effect that was attenuated by a direct but not an indirect FXa inhibitor and anticoagulant, which may have implications for clinical therapeutic responses.

SARS-CoV-2 is a newly emergent human pathogen that belongs to the beta-coronavirus containing a single-stranded RNA associated with a nucleoprotein within a capsid^32^. Unlike bats, which display immune tolerance, humans infected with SARS-CoV-2 sometimes over-activate inflammatory components of the immune system, triggering cytokine release syndrome^33^, which can be fatal in some people though nonexistent in others with the same exposure^34^. Therefore, identifying the body’s natural defense mechanisms against SARS-CoV-2 is important for understanding host susceptibility as well as for developing effective preventive and therapeutic strategies.

SARS-CoV-2 utilizes the ACE2 receptor to enter host cells. When its S protein is processed proteolytically by SPs such as TMPRSS2, furin, and trypsin, ACE2-mediated viral entry is enhanced^10,35^. Furin and TMPRSS2 cleave S protein at the S1/S2 site of at RRAR (685 R), followed by the S2’ site KPSKR (R856)^36,37^, resulting in priming and activating the SARS-CoV-2 fusion step. However, each SP has a different cleavage site and therefore affects viral entry into host cells differently from other family members. Thus, FXa cleaves S protein in a way that diminishes—rather than enhances—the S protein’s ability to interact with ACE2, thereby keeping the virus out of host cells. How cleavage of S protein by FXa blocks entry of SARS-CoV-2 into host cells is currently unknown. However, our in silico modeling, supported by experimental evidence, suggests that the cleavage sites of FXa on S protein are at Ile-(Asp/Glu)-Gly-Arg (R1000) or Gly-Arg (R567) and not at S1/S2 or S2’. Perhaps cleavage at those sites results in a unique conformational change in S protein. Moreover, the most conserved region of the RBD is between amino acids 319–541^38^, which is close to one of FXa’s cleavage sites (R567). Therefore, cleavage by FXa may adversely impact the RBD’s conserved conformation. S protein is also a type 1 viral fusion protein with two conserved heptad repeat regions, HR-N (916–950) and HR-C (1150–1185), which form a 6-helix bundle that enhances fusion between the virus and host cell membrane^21,39^. The predicted FXa cleavage site at 1000 is close to the HR-N and the HR-C repeat. However, whether cleavage at this site will affect the formation of the 6-helix bundle remains to be determined. In addition, the amino acid sequences of SARS-CoV-2 and SARS-CoV-1 are different, and this may result in opposite effects of FXa on the two viruses^40–42^.

Since the B.1.1.7 variant emerged in the United Kingdom in September 2020, it has demonstrated higher transmissibility^26,43^ and mortality^44^. Some vaccines and neutralizing antibodies are less effective against the B.1.1.7 variant than against the WT strain^45–48^. However, the reasons for the higher transmissibility and mortality of the B.1.1.7 variant remain elusive^49^. Our description of the previously unknown FXa response to SARS-CoV-2 infection and its differential effect on the B.1.1.7 variant is a possible, or at least partial, explanation. Our data showed that the B.1.1.7 variant, with its mutated S protein, was more resistant to inhibition by FXa in vitro and in vivo when compared to the WT strain. This should not be attributed to the recently identified loss of furin PRRAR cleavage site found in the B.1.1.7 variant that we used, as the loss of the site resulted in an attenuated variant^50^ and as other variants lacking this deletion, e.g., D614G and Delta, showed similar less efficiency in the FXa-mediated infection inhibition. Therefore, a host antiviral defense system depending on FXa might not be strong enough to protect humans from infection by the SARS-CoV-2 B.1.1.7 variant. The basis for the difference between variants could be the D614G mutation, which might also be responsible for the success of other variants such as Delta and Omicron. Of note, all pandemic variants identified to date carry the D614G mutation^51^. In agreement with our speculation, our data show that all the variants that we tested, including B.1.1.7, Delta, and Omicron as well as the engineered variant D614G + A570D contain the D614G mutation, are relatively resistant to cleavage of S protein by FXa, and are therefore more infectious than the WT strain at least in the presence of FXa. Moreover, others have reported that the D614G mutation in S protein increases SARS-CoV-2 infection of multiple human cell types and increases transmission rates^28,29^. One speculation is that this mutation may change the conformation of S protein, thereby affecting the S protein’s interaction with FXa.

FXa is required for the conversion of prothrombin to thrombin in the clotting cascade^13^, and may have a role in inflammation^52^. Both processes are dysregulated in some patients with COVID-19^34,53,54^. Many coagulation factors were important predictors of the clinical outcome in COVID-19 patients^54,55^. However, prior to our study, the role of FXa in viral infection has been unclear. Limited studies were based on theoretical assumptions rather than experimental data, or on SARS-CoV-1, which has different S protein sequences and thus different FXa cleavage sites compared to SARS-CoV-2, or on low concentrations of SARS-CoV-2 without pre-virus treatment to distinguish the mechanisms of action on the virus and host^15,35,40,56–60^. Prior to our study, no in vivo studies with live SARS-CoV-2 had been performed to elucidate the role of FXa in SARS-CoV-2 infection, as we pursued. Due to the complexity of clinical studies and the multiple roles of FXa in coagulation and host defense against infection, it is not surprising that there were inconsistent conclusions from two retrospective studies of COVID-19 as to whether anticoagulation increased or decreased mortality^61,62^. It should be noted that while both studies mentioned the use of direct (oral) FXa inhibitors for anticoagulation, patients were switched to indirect FXa inhibitors during hospitalization or during the acute phase of the illness. Based on the experimental evidence presented in this report, we believe that direct FXa inhibitors promote viral entry, thereby enhancing infectivity.

With extensive in vitro and in vivo studies—now including live SARS-CoV-2—we demonstrated that FXa cleaves S protein into non-S1 and S2 fragments, thus inhibiting infection by SARS-CoV-2 or VSV-SARS-CoV-2. By comparing other SPs in parallel and using dose gradients, we confirmed that furin, TMPRSS2, and trypsin facilitate—rather than impede—infection. We also demonstrated that direct inhibition of FXa abrogates the protein’s anti-SARS-CoV-2 activity, while indirect FXa inhibition does not. Moreover, administration of exogenous FXa during experimental SARS-CoV-2 infection produced antiviral activity in a dose-dependent manner. It has been reported that both liver and extrahepatic immune cells can produce FXa^63^. It will be interesting to explore whether different sources of FXa play different functions and whether FXa produced by pulmonary macrophages has anti-SARS-CoV-2 effect, based on the mechanism that we characterized in the current study.

Although the viral defense mechanism that we describe is likely to be important for controlling asymptomatic and mildly symptomatic SARS-CoV-2 infections, endogenous overexpression of FXa during severe SARS-CoV-2 infection might contribute to complications of COVID-19, especially thrombotic events^53,58,64^. Therefore, using FXa as a therapeutic agent is not straightforward, and administration of an indirect FXa inhibitor as an anticoagulant in combination with the FXa-Fc fusion protein could be considered in the setting of a clinical trial. There are four clinically approved direct FXa inhibitors, including rivaroxaban, apixaban, edoxaban as well as betrixaban) and one indirect FXa inhibitor fondaparinux for use as anti-thrombotic agents in patients with hypercoagulable states^65^. Our study showed that in the presence of a direct inhibitor of FXa, rivaroxaban, the anti-SARS-CoV-2 activity of FXa is adversely affected not by the binding of S protein but by the cleavage of S protein. Further, rivaroxaban completely abrogated the decrease in viral load and the protective effect against lethal SARS-CoV-2 infection conferred by exogenous FXa. Therefore, our findings suggest that testing direct FXa inhibitors, such as rivaroxaban, in patients highly susceptible to severe COVID-19^15^ should likely proceed with caution as such compounds could conceivably increase viral load. Likewise, patients who require an FXa inhibitor for chronic anticoagulation and are at high risk for clinically significant COVID-19 should likely be placed on an indirect FXa inhibitor outside of a prospectively randomized double-blind clinical trial. Importantly, the FXa indirect inhibitor fondaparinux, which we found did not block FXa’s protective effects against SARS-CoV-2, has proven to be safe and effective for venous thrombosis prophylaxis in hospitalized COVID-19 patients^22^.

In summary, we show that FX, the precursor of FXa, is upregulated in COVID-19 patients. We identify a mechanism of antiviral defense involving FXa in humans and demonstrate its protection against SARS-CoV-2 infection in vitro and in vivo with the K18-hACE2 animal model that mimics human disease. Accordingly, FXa-Fc can be developed as a therapeutic agent to treat COVID-19. Our experimental work suggests that when necessary, indirect FXa inhibitors should be considered over direct inhibitors when anticoagulation is indicated in COVID-19 patients.

## Methods

### Ethics statement

The protocols for human specimen collection were approved by the institutional review board of the City of Hope. Patients whose samples were investigated gave written informed consent. There was no bias in the selection of patients.

Experiments and handling of mice were conducted under federal, state, and local guidelines and with approvals from the Northern Arizona University and City of Hope Animal Care and Use Committees. Both female and male mice were used in this study, and no sex-based analysis was performed.

### Patient samples

Samples of patients tested positive for SARS-CoV-2 were collected at City of Hope. Autopsy samples were provided by Dr. Ross Zumwalt at the University of New Mexico School of Medicine. Concentrations of FXa in samples of patients’ plasma were measured using an ELISA kit (LifeSpan BioSciences, WA). Patient information is presented in Supplementary Table 1.

### Cells

Monkey kidney epithelial-derived MA104 cells were maintained in medium 199 supplemented with 10% FBS, penicillin (100 U/ml), and streptomycin (100 μg/ml). To overexpress FXa in MA104 cells, the cells were infected with lentivirus encoding FXa to generate MA104-FXa cells. Monkey kidney epithelium-derived Vero cells (Vero E6 cells), human embryonic kidney-derived HEK293T cells, Chinese hamster ovary (CHO) cells, Calu3 cells, and adenocarcinomic human alveolar basal epithelial cells A549 were cultured in DMEM with 10% FBS, penicillin (100 U/ml), and streptomycin (100 μg/ml). Vero cells were obtained from the laboratory of E. Antonio Chiocca. MA104 cells were obtained from the laboratory of Dr. Sean P.J. Whelan at Washington University School of Medicine in St. Louis. The rest of the cell lines were purchased from the American Type Culture Collection (ATCC). All cell lines were routinely tested to confirm the absence of mycoplasma, using the MycoAlert Plus Mycoplasma Detection Kit from Lonza (Walkersville, MD).

### VSV-SARS-CoV-2 infection

The chimeric VSV-SARS-CoV-2 virus expressing GFP was kindly provided by Dr. Sean Whelan. The virus is decorated with SARS-CoV-2 S protein in place of its native glycoprotein (G)^19^. For VSV-SARS-CoV-2 infection, MA104 cells were seeded 24 hours before the infection at a confluency of 70% in a 96-well plate. VSV-SARS-CoV-2 virus and varying amounts (as indicated in each figure, e.g., 16 nM, 32 nM, 62.5 nM, 100 nM, 125 nM, 250 nM, 500 nM, and 1 µM) of the FXa-Fc fusion protein were co-incubated at 37°C for 1 hour and then added to the cells. For assessing the effect of FXa inhibitors, FXa protein was preincubated with or without 50 µg/ml rivaroxaban or fondaparinux for 1 hour at room temperature. Infectivity was assessed by detecting GFP fluorescence using a Zeiss fluorescence microscope (AXIO observer 7) and/or determined by the percentage of GFP(+) cells analyzed with a Fortessa X20 flow cytometer (BD Biosciences) at 16, 24, 36, and 48 hours post infection (hpi). For determination of virus production, Vero cells were pre-seeded for 24 hours and infected with supernatants collected from MA104 cells infected with VSV-SARS-CoV-2 at 24 or 48 hpi. The supernatants were diluted 5-fold before the viral production assay.

### Generation and purification of FXa-Fc

CHO cells were transduced with a pCDH lentiviral vector expressing FXa to produce the FXa-Fc fusion protein for functionality assays. For this purpose, FXa fused with human IgG4 was reconstructed and inserted into the pCDH vector^66^. mCherry was co-expressed with FXa for FACS-sorting to purify transduced cells using a FACS Aria II cell sorter (BD Biosciences, San Jose, CA, USA). Conditional supernatants from lentivirus-infected CHO cells sorted by FACS were used to purify the FXa-Fc fusion protein on a protein G column (89927, Thermo Fisher). For in vivo testing, FXa-Fc fusion protein from the column was desalted using fast protein liquid chromatography (FPLC).

### SARS-CoV-2 cell infection and immuno-plaque assay

The following reagents were obtained through BEI Resources, NIAID, NIH: SARS-Related Coronavirus 2, Isolate USA-WA1/2020, NR-52281 (WT) and SARS-Related Coronavirus 2, Isolate USA/CA_CDC_5574/2020, NR-54011 (B.1.1.7). SARS-Related Coronavirus 2 isolate TG898390, B.1.617.2 (Delta) was kindly provided by Dr. Pei-Yong Shi (University of Texas Medical Branch) and the World Reference Center for Emerging Viruses and Arboviruses (WRCEVA). These recombinant isolates were previously described^29,30^. Virus isolates were passaged in Vero E6 cells (ATCC CRL-1586) or Calu3 cells (ATCC HTB-55), as previously described^67^. Virus concentrations were determined using immuno-plaque assay (also called focus forming assay)^68^. For the assay, indicated concentrations of FXa were pre-treated with different MOIs of live SARS-CoV-2 variants for 1 hour or concomitantly treated with the virus; then the mixture was added to Vero E6 cells, MA104 cells, or A549-ACE2 cells for 1 hour at 37 °C. The medium containing virus was then removed, overlaid with medium containing methylcellulose and 2% FBS DMEM, and incubated at 37°C. At 24-36 hours after infection, infected cells were fixed with 4% paraformaldehyde for 20 minutes at room temperature and then permeabilized in 0.5% Triton X-100/PBS solution for 210 minutes at room temperature. SARS-CoV-2 viral nucleocapsid protein (NP) was detected using the anti-NP protein antibody (PA5-81794, Thermo Fisher) diluted 1:10000 in 0.1% tween-20/1% BSA/PBS solution as a primary antibody. Detection with an anti-rabbit secondary antibody (ab6721, Abcam) at a 1:20,000 dilution followed. Plates were washed three times between antibody solutions, using 0.5% tween-20 in PBS. The plates were developed using TrueBlue Peroxidase Substrate (5510-0030, Sera Care) and then scanned using Immunospot S6 Sentry (C.T.L Analyzers).

### ELISA assessment of binding between FXa and S protein or VSV-SARS-CoV-2 viral particles

Coronavirus S protein with His tag consisting of S1 and S2 extracellular domain produced in baculovirus-insect cells (500 ng, 40589-V08B1, Sino Biological), coronavirus S protein S1 subunit with His tag (500 ng,40591-V08B1, Sino Biological), coronavirus S protein S2 subunit with His tag (500 ng, 40070-V08B, Sino Biological), coronavirus S protein RBD with His tag (500 ng, 40592-V08B-B, Sino Biological), and 10^4^ PFU VSV-SARS-CoV-2 viral particles were used as coating reagents in 96-well plates (3361, Corning). Coated plates were incubated with FXa protein (1 µg/ml) for 2 hours at room temperature. HRP-conjugated anti-human Fc antibody (05-4220, Invitrogen) at a 1:1000 dilution was used as a detecting antibody. Absorbance was measured at OD 450 nm, using a Multiskan™ FC Microplate Photometer (Fisher Scientific).

### Pull-down assay

HEK293T cells were transduced for 48 hours with a pCDH lentiviral vector expressing full-length S protein. The cells were lysed and incubated with FXa-Fc or Fc (10 µg/ml) for 3 hours, then 20 µl protein A agarose resin beads (P-400-25, Invitrogen) were added, and the mixture was incubated overnight. After the incubation, the beads were washed and collected. Binding between FXa-Fc or Fc protein and S protein was detected by immunoblotting, using an anti-S protein antibody (ab272504, Abcam) at a 1:1000 dilution.

### Cleavage assay

One microgram of the above S protein (S1 plus S2 full-length extracellular domain) was treated with 1 µg of FXa (P8010L, NEB), furin (P8077S, NEB), or TMPRSS2 (TMPRSS2-1856H, Creative BioMart) protein for 3 hours following the manufacturers’ instructions. Cleavage was detected using immunoblotting with an anti-S protein antibody (40591-T62, Sino Biological) at a 1:1000 dilution. For the cleavage assay using VSV-SARS-CoV-2 viral particles, 5 × 10^4^ PFU VSV-SARS-CoV-2 viral particles were treated with 1 µg of FXa or furin protein for 3 hours following the manufacturers’ instructions. Cleavage was detected using immunoblotting with an anti-S protein antibody (40591-T62, Sino Biological) at a 1:1000 dilution. For evaluating the cleavage fragments, anti-RBD antibody (MAB10540-100, R&D) and anti-S2 subunit antibody (MA5-35946, Invitrogen) were used at the 1:1000 dilution. For inhibitor assays, FXa protein was preincubated with or without 50 µg/ml rivaroxaban or fondaparinux separately for 1 hour at room temperature and then treated with SPs for 3 hours, followed by the detection procedure as described above. For cleavage assays using S protein–ACE2 complex, 1 µg S protein and 1 µg ACE2 were pre-incubated 1 hour prior to incubation with FXa, then treated with SPs and detected as above. Of note, we used different buffer conditions for all binding assays and cleavage assays. For comparing the cleavage efficiency between S protein of different variants, different S protein was treated with 1 µg of FXa for a shorter time period of 1 hour instead of 3 hours and detected as above.

### Liquid chromatography-mass spectrometry (LC-MS)/MS analysis

LC-MS/MS analysis was performed with an EASY-nLC 1200 (ThermoFisher Scientific, San Jose, CA) coupled to an Orbitrap Eclipse Tribrid mass spectrometerer (ThermoFisher Scientific, San Jose, CA). Peptides were separated on an Aurora UHPLC Column (25 cm × 75 μm, 1.6 μm C18, AUR2-25075C18A, Ion Opticks) with a flow rate of 0.35 μL/min for a total duration of 135 min and ionized at 1.6 kV in the positive ion mode. The gradient was composed of 6% solvent B (7.5 min), 6–25% B (82.5 min), 25–40% B (30 min), and 40–98% B (15 min). Solvent A is 0.1% formic acid in water, and solvent B is 80% acetonitrile (CAN) and 0.1% formic acid. MS1 scans were acquired at the resolution of 120,000 from 350 to 2000 m/z, automatic gain control (AGC) target 1 × 10^6^, and maximum injection time 50 ms. MS2 scans were acquired in the ion trap using fast scan rate on precursors with 2–7 charge states and quadrupole isolation mode (isolation window: 0.7 m/z) with higher-energy collisional dissociation (HCD, 30%) activation type. Dynamic exclusion was set to 30 s. The temperature of the ion transfer tube was 300 °C and the S-lens RF level was set to 30. MS2 fragmentation spectra were searched with Proteome Discoverer SEQUEST (version 2.5, Thermo Scientific) against in silico tryptic digested Uniprot SARS-COV-2 Spike database. The maximum missed cleavages were set to 2. Dynamic modifications were set to oxidation on methionine (M, +15.995 Da), phosphoribosylation (D, E, R, and K, +212.009 Da), deamidation (N and Q, +0.984 Da), protein N-terminal acetylation (+42.011 Da) and Met-loss (−131.040 Da). Carbamidomethylation on cysteine residues (C, +57.021 Da) was set as a fixed modification. The maximum parental mass error was set to 10 ppm, and the MS2 mass tolerance was set to 0.6 Da. The false discovery threshold was set strictly to 0.01 using the Percolator Node validated by q-value. The relative abundance of parental peptides was calculated by integration of the area under the curve of the MS1 peaks using the Minora LFQ node.

### Assessing binding between S protein and FXa with flow cytometry

HEK293T cells were transduced for 48 hours with a lentiviral vector expressing membrane-bound FXa displayed by a human PDGFRβ transmembrane domain. The cells were then incubated with 10 µg/ml full-length S protein for 20 minutes at room temperature. They were then washed and incubated with an anti-S protein antibody for 20 minutes at room temperature and stained with a FITC-labeled secondary antibody (111-605-045, Jackson ImmunoResearch) at a 1:1000 dilution. The percentage of FITC-positive cells was determined using a Fortessa X20 flow cytometer (BD Biosciences). Flow cytometry gating strategy is shown in Supplementary Fig. 14.

### Detection of FXa binding to the S protein–ACE2 complex, using ELISA

ACE2 protein was used as a coating reagent in 96-well plates, which were incubated with 1 µg/ml full-length S protein with His tag pre-treated with or without FXa (P8010L, NEB) for 2 hours at room temperature. An HRP-conjugated anti-His tag antibody (ab1187, Abcam) at a 1:1000 dilution was the detecting antibody. Absorbance was measured at OD 450 nm, using a Multiskan™ FC Microplate Photometer (Fisher Scientific).

### Detection of FXa binding to the S protein–ACE2 complex using flow cytometry

HEK293T cells stably expressing ACE2 protein were incubated with full-length S protein or full-length S protein pre-treated with FXa for 20 minutes at room temperature. Cells were then washed and incubated with an anti-S protein antibody for 20 minutes at room temperature and then stained with an APC-labeled secondary antibody (111-005-003, Jackson ImmunoResearch) at a 1:1000 dilution. The percentage of APC-positive cells was determined using a Fortessa X20 flow cytometer (BD Biosciences).

### In vivo infection model

6–8-week-old male and female K18-hACE2 mice in the C57BL/6 J background were anesthetized with ketamine (80 mg/kg)/xylazine (8 mg/kg) and intranasally infected with 5 × 10^3^ PFU WT SARS-CoV-2 or B.1.1.7 variant in 25 µl DMEM, followed by intranasal treatment with PBS, FXa-Fc (200 µg), FXa-Fc (2 µg), or Fc (200 µg) in 25 µl DMEM. Infected mice were maintained in bio-containment unit isolator cages (Allentown, NJ, USA) in the NAU ABLS3. Mice were then treated with PBS or rivaroxaban (30 mg/kg) via gavage or fondaparinux (30 mg/kg) via intraperitoneal injection four times at a frequency of every other day. The body weights of mice were monitored daily. Mice were euthanized using ketamine (100 mg/kg)/xylazine (10 mg/kg) when body weights dropped below 20% of their original body weights. RNA was isolated from trachea, lung, and brain tissues to assess viral load using quantitative real-time PCR as described below. Expression of SARS-CoV-2 viral protein NP was examined using immunohistochemistry (IHC) in the trachea, lung, and brain sections of infected mice, as described below.

We generated FX knockout C57BL/6 J mice by CRISPR/Cas9 gene-editing technology. We crossed the mice with K18-hACE2 mice and obtained the FX (heterozygotes)-K18-hACE2 strain. We inoculated 6–8-week-old male and female FX(heterozygotes)-K18-hACE2 mice and K18-hACE2 mice with 5 × 10^3^ PFU SARS-CoV-2 at day 0. On day 5, mice were euthanized, and lung tissues were collected to measure viral load, as described below.

### Quantitative real-time PCR

Mouse tissues were homogenized in DMEM, and RNA was isolated using a PureLink RNA isolation kit (K156002, Invitrogen). mRNA levels of S protein, FX expression, and 18 S ribosomal (r) RNA expression were determined with the One-Step qPCR kit (1725150, BioRad). The primer sequences were CoV-2-S Forward (5’-GCTGAACATGTCAACAACTC-3’), CoV2-S Reverse (5’-GCAATGATGGATTGACTAGC-3’), FX Forward (5’-AGGACTCGGAGGGCAAACT-3’), FX Reverse (5’-TCACGGACCTCTTCATAAGAACA-3’), 18 S rRNA Forward (5’-GTAACCCGTTGAACCCCATT-3’) and 18 S rRNA Reverse (5’- CCATCCAATCGGTAGTAGCG-3’).

### H&E and IHC

4-μm-thick sections were cut from paraffin blocks of lung and liver tissues from COVID-19 patients and non-COVID-19 donors. The following primary antibodies were used for IHC staining: an anti-FXa protein antibody (PIPA529118, Invitrogen, at a 1:500 dilution), an anti-furin antibody (ab183495, Abcam, at a 1:500 dilution), an anti-trypsin antibody (ab200997, Abcam, at a 1:500 dilution), or an anti-plasmin antibody (LS-C150813-1, LSBio, at a 1:500 dilution). IHC was performed by the Pathology Shared Resource Core at City of Hope Beckman Research Institute. Stained slides were mounted and scanned for observation.

Tissues isolated from the experimental mice were placed in 10% neutral buffered formalin for a minimum of 72 hours. After paraffin embedding, 4-μm-thick sections were cut from the blocks. H&E staining and IHC with an anti-NP protein antibody (NB100-56576, Novus, at a 1:500 dilution) as the primary antibody were performed by the Pathology Shared Resource Core at City of Hope Beckman Research Institute. Stained slides were mounted and scanned for observation.

### Statistical analysis

Prism software v.8 (GraphPad, CA, USA) and SAS v.9.4 (SAS Institute. NC, USA) were used to perform statistical analyses. Data were summarized by descriptive statistics (mean, SD, count, etc.). For measures following a relatively symmetric distribution while without significant departures from the normal distribution, Student’s *t* test was used to compare two independent groups. One-way ANOVA models were used to compare three or more independent groups. Two-way ANOVA models were used to assess interactions between two factors, such as treatment by time or treatment by MOI. Data transformations (e.g., log base 2) were applied to the measures that ranged on a wide scale and suggested a potential trend of skewed distributions, such as MFI and copy number in some experiments. Graphical methods were employed to assess the trend of change over time for repeated measures such as body weight. Survival functions were estimated using the Kaplan–Meier method and compared using the two-sided log-rank test. All tests were two-sided. *P* values were adjusted for multiple comparisons using Hochberg’s method. A *P* value of 0.05 or less was considered statistically significant.

### Reporting summary

Further information on research design is available in the Nature Portfolio Reporting Summary linked to this article.

## Supplementary information

Supplementary Information

Description of Additional Supplementary Files

Supplementary Data 1

Reporting Summary

## Data Availability

Uniprot SARS-COV-2 Spike database (https://covid-19.uniprot.org/uniprotkb/P0DTC2) was used as a reference for liquid chromatography mass spectrometry data. Source data for Figs. 1–7 and Supplementary Figs. 1–13 has been provided as Source Data in this paper. All other data supporting the findings of this study are available from the corresponding author upon reasonable request. Source data are provided in this paper.

## Source data

Source Data
