# Supplementary material for: Factor Xa cleaves SARS-CoV-2 spike protein to block viral entry and infection
Source: Nat Commun. 2023 Apr 6;14:1936. doi: 10.1038/s41467-023-37336-9 (PMC10079155; doi:10.1038/s41467-023-37336-9)
Supplement: Supplementary file 1 — Supplementary Information [file 41467_2023_37336_MOESM1_ESM.pdf]

Supplementary Information

Factor Xa cleaves SARS-CoV-2 spike protein to block viral entry and infection

Wenjuan Dong[1,2,†], Jing Wang[1,2,†], Lei Tian[1,2,†], Jianying Zhang[3], Erik W. Settles[4,5], Chao Qin[6], Daniel R. Steinken- Kollath[4], Ashley N. Itogawa[4], Kimberly R. Celona[4], Jinhee Yi[4], Mitchell Bryant[4], Heather Mead[4], Sierra A. Jaramillo[4], Hongjia Lu[7], Aimin Li[8], Ross E. Zumwalt[9], Sanjeet Dadwal[10], Pinghui Feng[6], Weiming Yuan[7], Sean P.J. Whelan[11], Paul S. Keim[4,5], Bridget Marie Barker[4,5], Michael A. Caligiuri[1,2,12*], and Jianhua Yu[1,2,12,13,*]

[1]Department of Hematology & Hematopoietic Cell Transplantation, City of Hope National Medical Center, Los Angeles, CA 91010, USA

[2]Hematologic Malignancies Research Institute, City of Hope National Medical Center, Los Angeles, CA 91010, USA

[3]Department of Computational and Quantitative Medicine, City of Hope National Medical Center, Los Angeles, CA 91010, USA

[4]Pathogen and Microbiome Institute, Northern Arizona University, Flagstaff, AZ 86011, USA

[5]Department of Biological Sciences, Northern Arizona University, Flagstaff, AZ, 86011, USA

[6]Section of Infection and Immunity, Herman Ostrow School of Dentistry, Norris Comprehensive Cancer Center, University of Southern California, Los Angeles, CA 90089

[7]Department of Molecular Microbiology and Immunology, Keck School of Medicine of University of Southern California, Los Angeles, CA 90033, USA

[8]Pathology Core of Shared Resources Core, Beckman Research Institute, City of Hope National Medical Center, Los Angeles, CA 91010, USA

[9]Department of Pathology, University of New Mexico, Albuquerque, NM 87131, USA

[10]Division of Infectious Diseases, Department of Medicine, City of Hope National Medical Center, Los Angeles, CA 91010, USA

[11]Department of Molecular Microbiology, Washington University School of Medicine, St. Louis, MO 63110, USA

[12]City of Hope Comprehensive Cancer Center, Los Angeles, CA 91010, USA

[13]Department of Immuno-Oncology, City of Hope, Los Angeles, CA 91010, USA

[†]These authors contributed equally

[*]Correspondence should be addressed to Jianhua Yu, PhD, jiayu@coh.org; Michael A. Caligiuri, MD, mcaligiuri@coh.org

Short title: FXa cleaves spike and blocks SARS-CoV-2

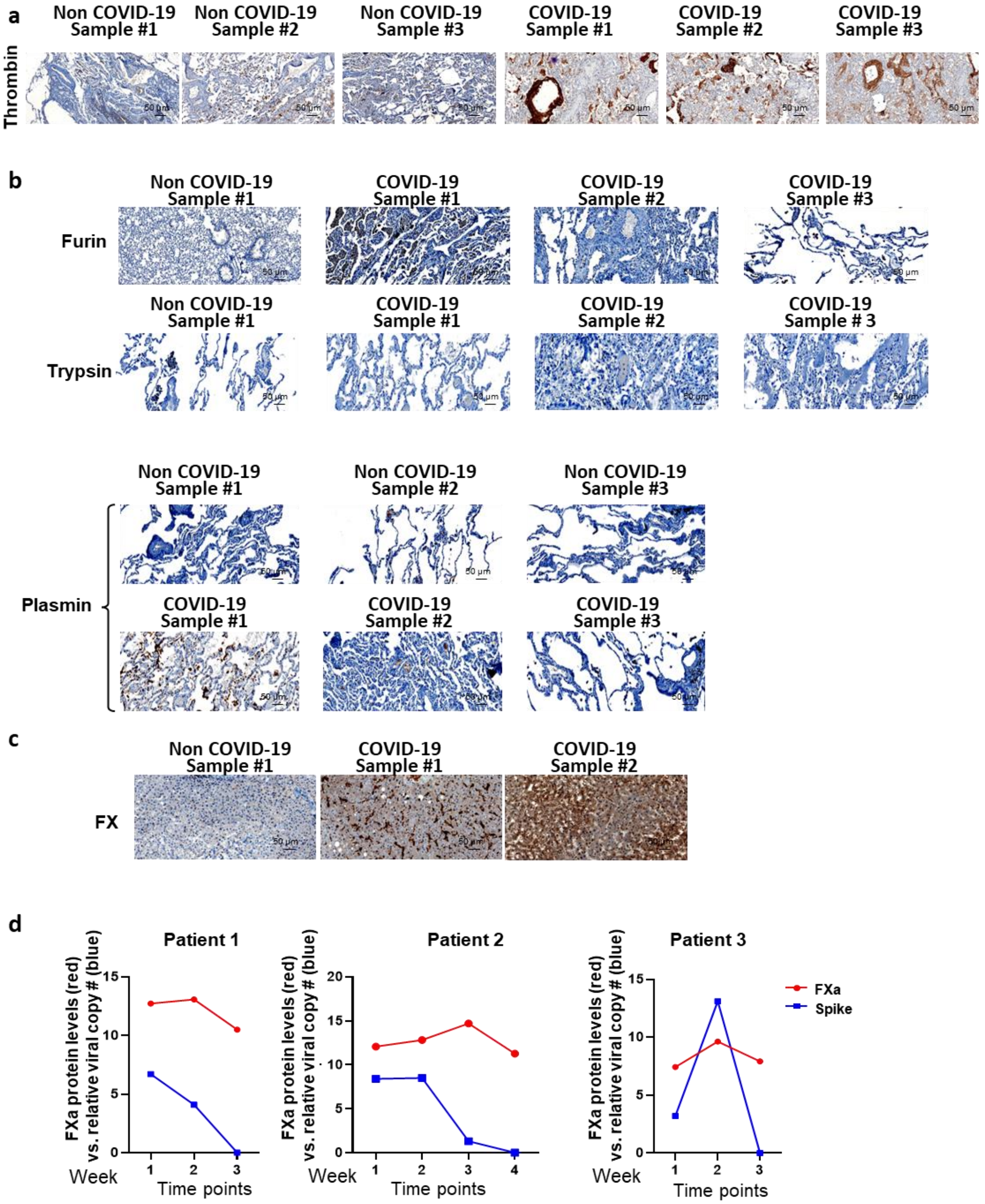

Supplementary Figure 1. Expression of serine proteases in autopsied organs of COVID-19 patients (**a**) Expression of thrombin in autopsied lung of patients who died of COVID-19 versus non-COVID-19 donors. **(b)** Expression of furin, trypsin, and plasmin in autopsied lung of the patients versus non-COVID-19 donors. **(c)** Expression of FX in autopsied liver of the patients versus non-COVID-19 donors. (**d**) FXa levels (µg/ml) in plasma as measured by ELISA (red) and spike expression in patients' nasopharyngeal swabs as measured by qPCR with normalization (blue) at different time points post diagnosis of COVID-19. In a-c, the staining results shown are representative of at least two independent experiments with similar results. Scale bar, 50 µm. d, n = 3 COVID-19 patients. Source data are provided as a Source Data file.

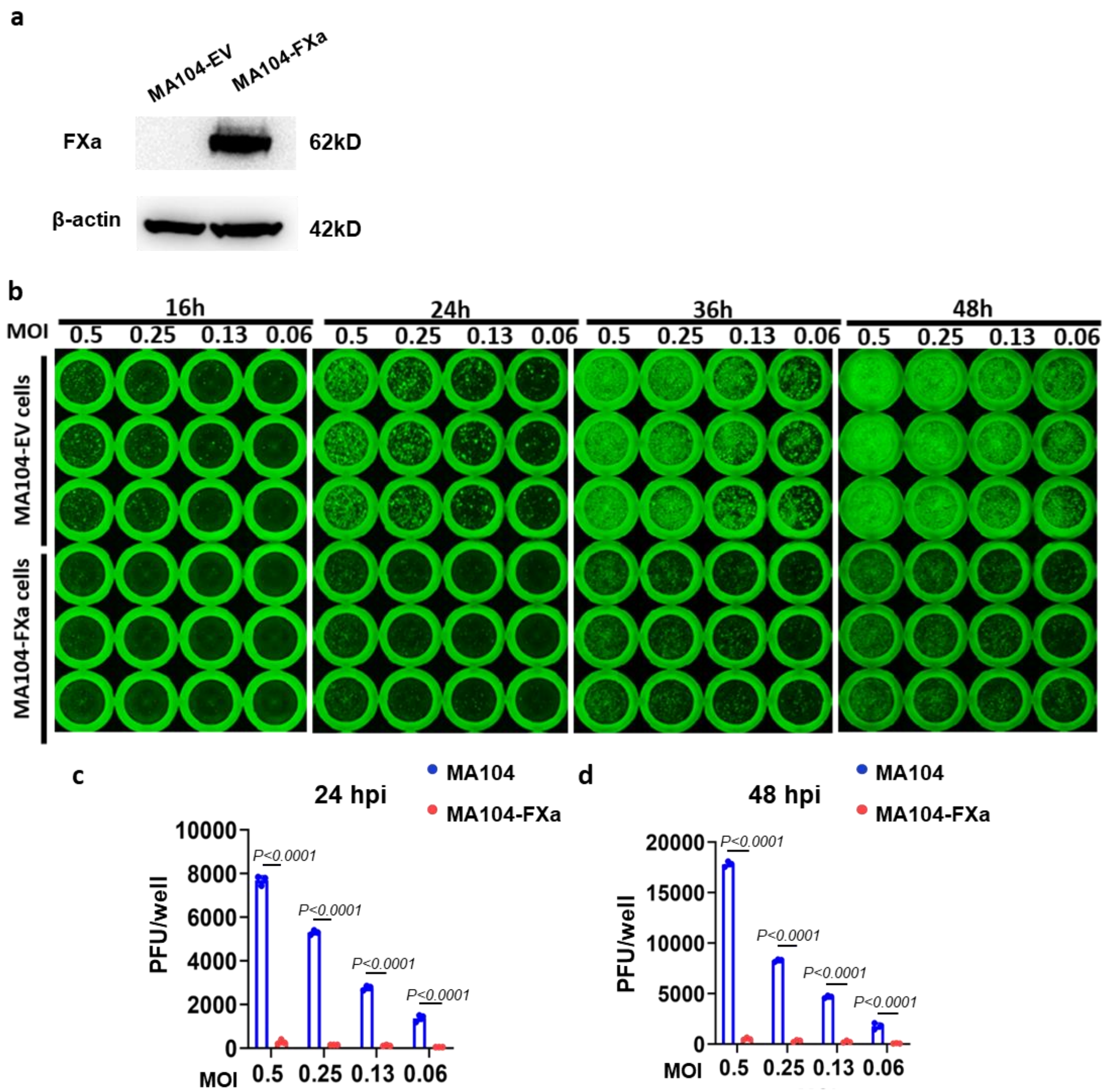

Supplementary Figure 2. Infectivity of VSV-SARS-CoV-2 virus in MA104 cells expressing FXa or a control vector. (**a**) Confirmation of forced overexpression of FXa in MA104 cells by immunoblotting. (**b**) MA104 cells transduced with FXa (MA104-FXa) or EV (MA104-EV) were infected with VSV-SARS-CoV-2 and imaged at 16, 24, 36, and 48 hpi by fluorescence microscopy. (**c** and **d**) Viral titer in supernatant from VSV-SARS-CoV-2-infected MA104 or MA104-FXa cells at 24 hpi (c) and 48 hpi (d) was determined by subsequently infecting Vero cells. All data are representative of three independent experiments. Data in c and d are presented as mean values ± SD and statistical analyses were performed by two-way ANOVA. PFU data were log2 transformed before running statistical models. Source data are provided as a Source Data file.

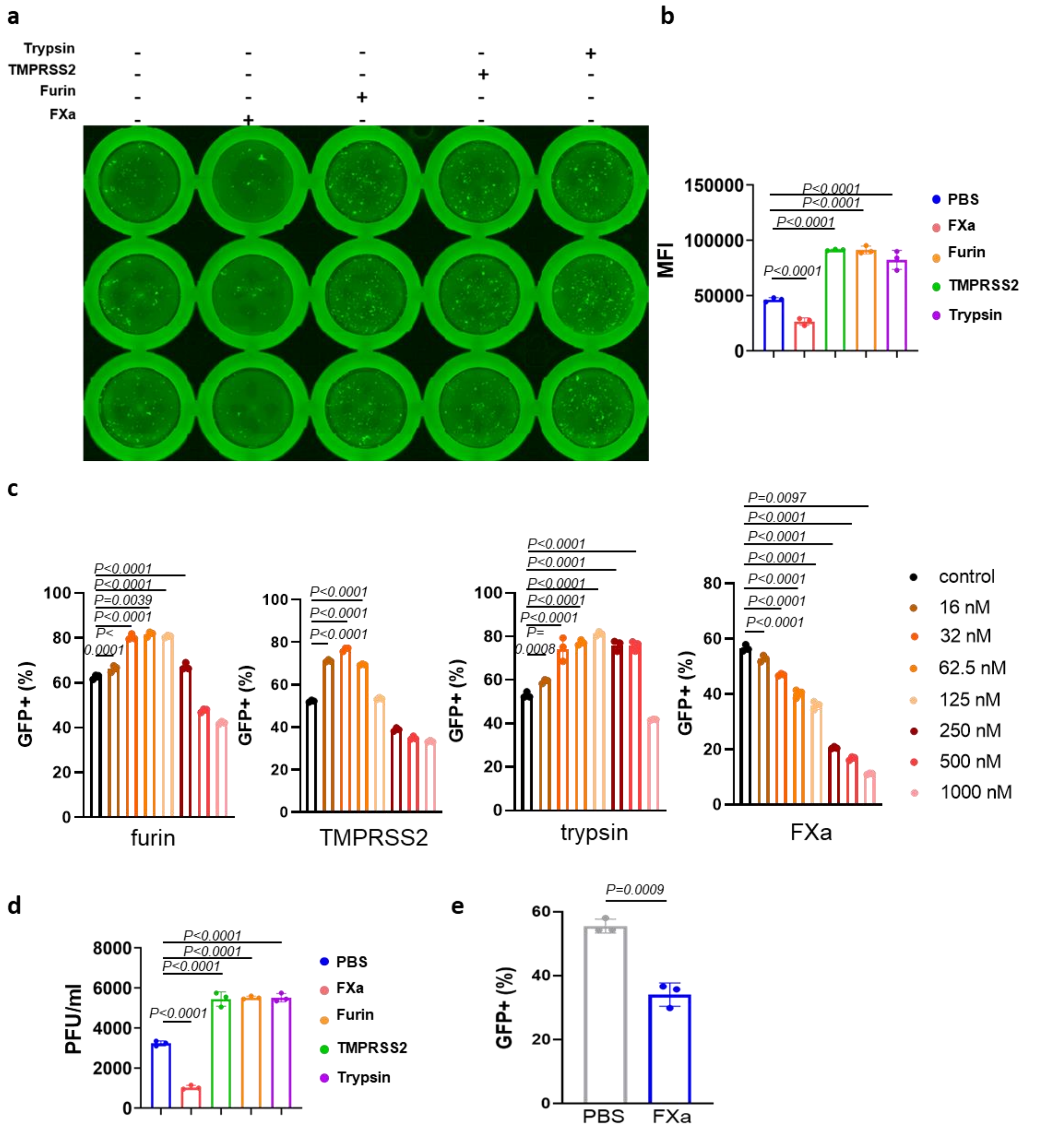

Supplementary Figure 3. FXa inhibits VSV-SARS-CoV-2 infection in MA104 cells while TMPRSS2, trypsin, and furin promote infection. (**a** and **b**) Infectivity of VSV-SARS-CoV-2 in MA104 cells in the presence or absence of FXa, TMPRSS2, trypsin, or furin, determined by fluorescence microscopy. (**c**) VSV-SARS-CoV-2 was preincubated with furin, TMPRSS2, trypsin, or FXa at the indicated concentrations for 1 hour before it was used to infect M104 cells. Infectivity was quantified by flow cytometry at 48 hpi. (**d**) Virus titration by MA104 cells infected with VSV-SARS-CoV-2 in the presence or absence of FXa, TMPRSS2, trypsin, or furin was determined by subsequently infecting Vero cells. (**e**) VSV-SARS-CoV-2 was preincubated with MA104 cells for 1 hour, washed twice, and then exposed to treatment with FXa. Infectivity of the cells was quantified by flow cytometry at 24 hpi. All data are representative of three independent experiments. Data in b-e are presented as mean values ± SD and statistical analyses were performed two-sided Student's t test (e) or one-way ANOVA (b-d). MFI (b) and PFU (d) data were log_2 transformed before running statistical models. Source data are provided as a Source Data file.

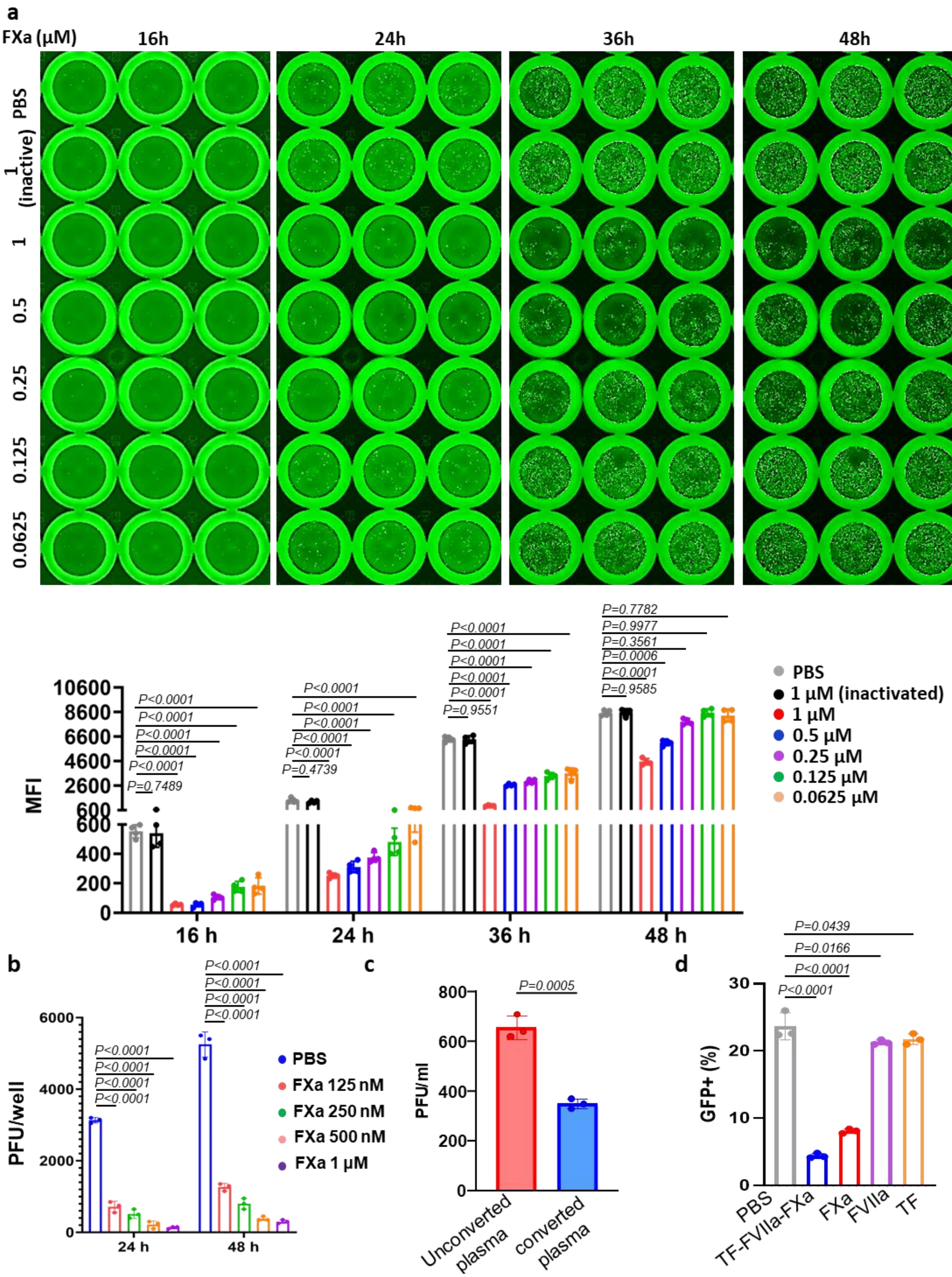

Supplementary Figure 4. FXa recombinant protein, its conversion from plasma, and TF-FVIIa-FXa complex inhibit VSV-SARS-CoV-2 infection in MA104 cells. (**a**) VSV-SARS-CoV-2 was preincubated with different concentrations of FXa for 1 hour before infecting M104 cells. Cells were imaged at 16, 24, 36, and 48 hpi by fluorescence microscopy (top panel), and the corresponding infectivity was measured by flow cytometry (bottom panel). N=4 independent experiments. (**b**) MA104 cells were infected with VSV-SARS-CoV-2 that had been preincubated with different concentrations of FXa for 1 hour.

Supernatants collected at 24 and 48 hpi were used to infect Vero cells for a virus titration assay. N=3 independent experiments. (**c**) VSV-SARS-CoV-2 was preincubated with plasma in which FX was converted or unconverted to FXa by incubating with FIXa and Factor V Activating Enzyme from Russell′s viper venom. Infectivity was measured at 24 hpi by fluorescence microscopy. N=3 independent experiments. (**d**) VSV-SARS-CoV-2 was preincubated with PBS, TF-FVIIa-FXa complex, FXa, FVIIa, or TF, followed by infecting M104 cells. Corresponding infectivity was measured by flow cytometry. N=3 independent experiments. Data are presented as mean values ± SD and statistical analyses were performed by two-sided Student's t test (c), one-way ANOVA (d), or two-way ANOVA (a, b). MFI (a) and PFU (b-c) data were $\log_2$ transformed before running statistical models. Source data are provided as a Source Data file.

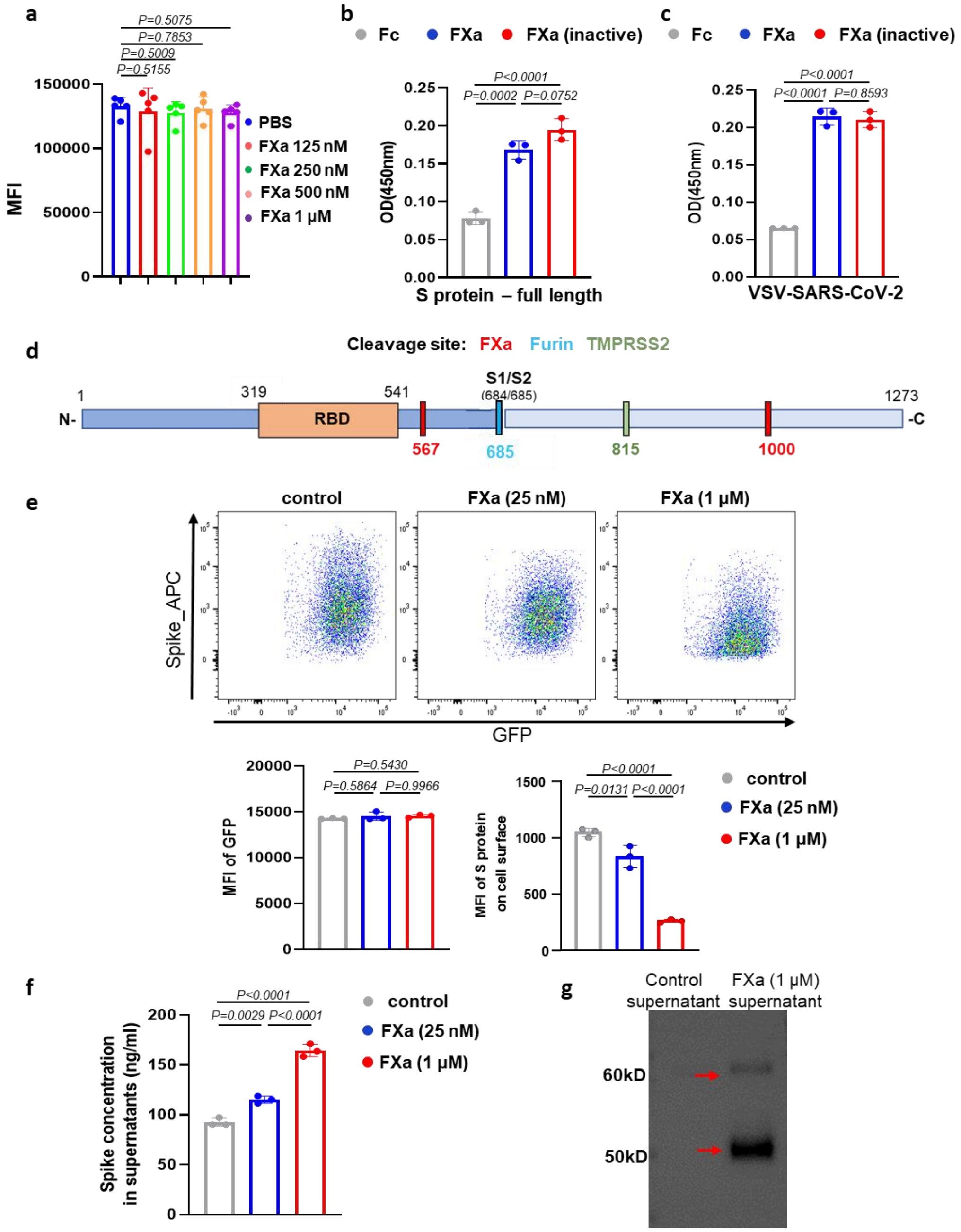

Supplementary Figure 5. FXa does not act on host cells but cleaves S protein expressed on cell surface, leading to shedding of S protein. (a) MA104 cells were incubated with or without FXa for 1 hr, washed, and then infected with VSV-SARS-CoV-2 for 24 hr. Viral infectivity was measured by flow cytometry, measuring the expression level of GFP from virally infected cells. N=5 independent experiments. **(b-c)** The binding affinity of active FXa-Fc, inactive FXa-Fc, and Fc to full-length S protein (b) or VSV-SARS-CoV-2 chimeric viral particles (c) was quantified by ELISA. N=3 biologically independent samples. **(d)** The *in silico* prediction of the sites in the full-length S protein cleaved by furin, TMPRSS2, or FXa. Dark and light blue shades represent S1 and S2 subunits of the spike protein, respectively. **(e-g)** Shedding S protein by FXa. GFP and S protein surface expression on A549 cells, referred to as A549-S cells, treated with or without FXa were detected by flow cytometry (e). S protein from the supernatants of FXa-treated A549-S cells was measured by ELISA (f) and immunoblotting assay

with an anti-S protein antibody (40591-T62, Sino Biological) (g). N=3 biologically independent samples. Data in g are representative of two independent experiments with similar data. Data are presented as mean values ± SD and statistical analyses were performed by one-way ANOVA. MFI data were log_2 transformed before running statistical models. Source data are provided as a Source Data file.

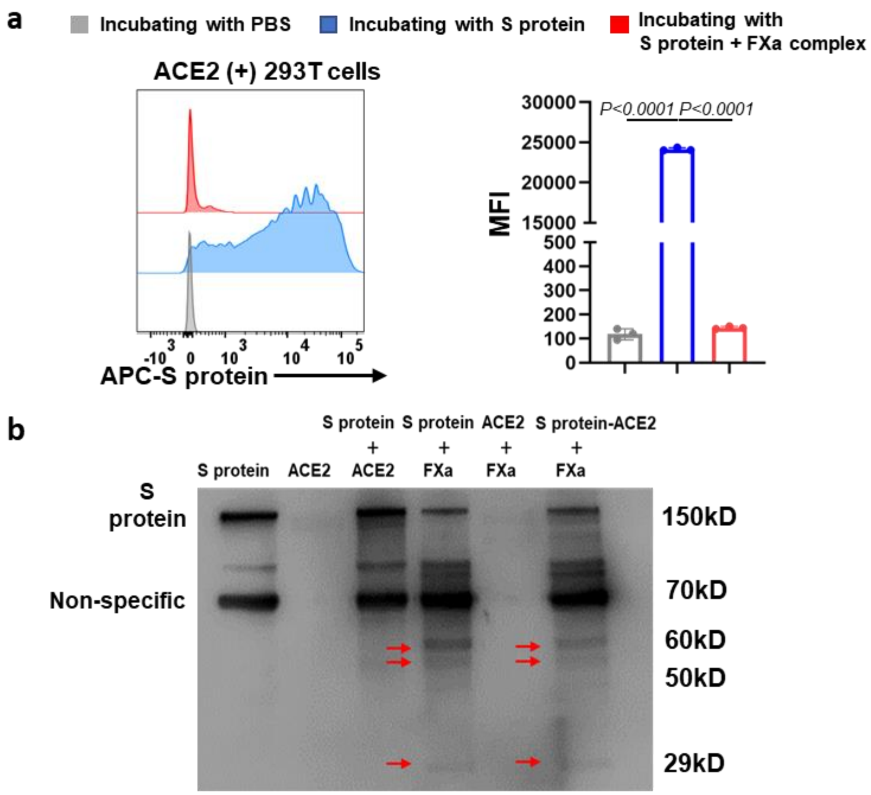

Supplementary Figure 6. FXa cleavage blocks binding between S protein and ACE2. (**a**) Binding of S protein or FXa-pretreated S protein to the cell surface ACE2, determined by the amount of S protein on 293T cells expressing ACE2 using flow cytometry (left, a representative flow cytometry histogram; right, summary data). N=3 biologically independent samples. Data are presented as mean values ± SD and statistical analysis was performed by one-way ANOVA. MFI data were $\log_2$ transformed before running statistical models. (**b**) S protein was incubated with ACE2 for 1 hour, and then FXa was added to the incubation for another hour. Cleavage of S protein in the S protein-ACE2 complex by FXa was determined by immunoblotting with an anti-S protein antibody (40591-T62, Sino Biological). Data are representative of three independent experiments. Source data are provided as a Source Data file.

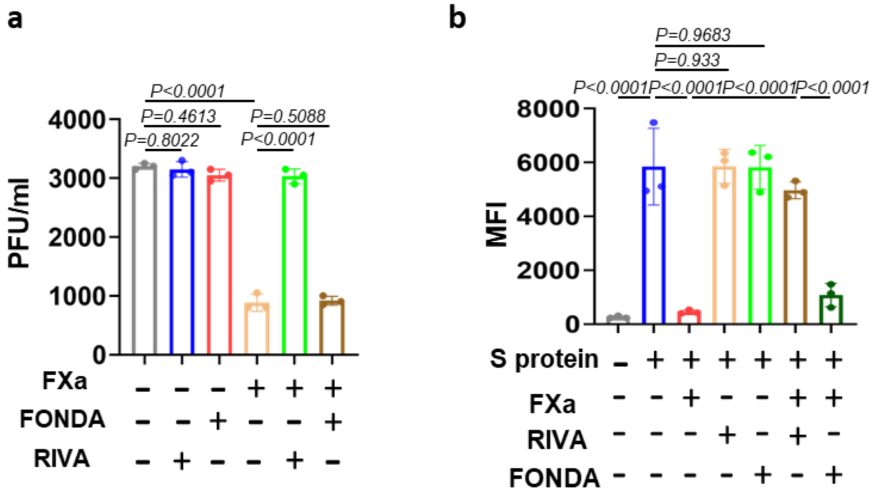

Supplementary Figure 7. The effect of direct and indirect FXa inhibitors on FXa-mediated inhibition of SARS-CoV-2. (a) Virus titration of FXa-pretreated vs. untreated VSV-SARS-CoV-2 in MA104 cells in the presence or absence of RIVA or FONDA was determined by re-infection of Vero cells with supernatants from MA104 infection. N=3 independent experiments. (**b**) S protein was incubated with FXa that was pretreated with or without RIVA or FONDA. The binding capability of these treated S proteins with ACE2 expressed on 293T cells was assessed by flow cytometry (summary data of main Fig. 3l). N=3 independent experiments. Data are presented as mean values ± SD and statistical analyses were performed by one-way ANOVA (a, b). Source data are provided as a Source Data file.

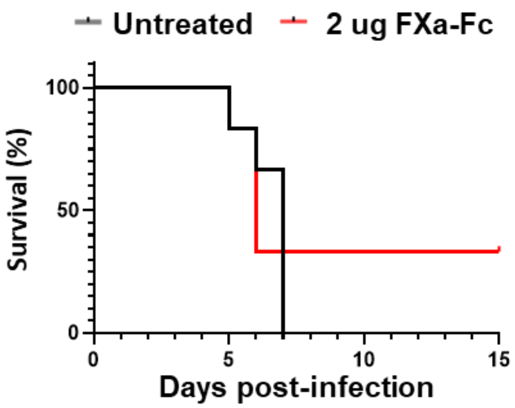

Supplementary Figure 8. The effect of low-dose on *in vivo* live SARS-CoV-2 infection. The survival of K18-hACE2 mice infected intranasally with 5×10^3 PFU SARS-CoV-2 WA1 strain treated intranasally with or without 2 μg FXa-Fc fusion protein per mouse (n=6 mice/group). Source data are provided as a Source Data file.

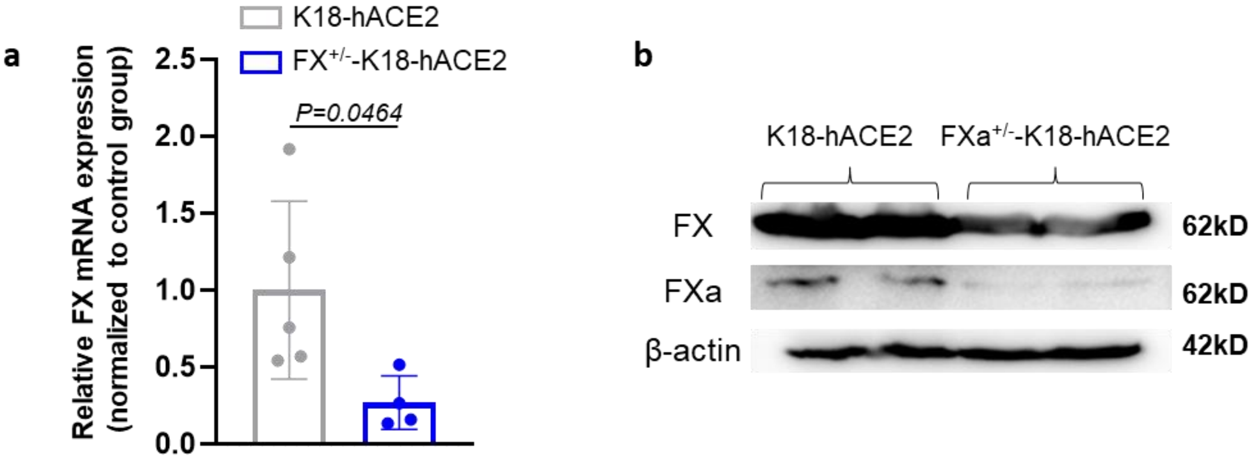

Supplementary Figure 9. The effect of endogenous of FXa on *in vivo* live SARS-CoV-2 infection. (**a** and **b**) Expression of FXa in lungs of $FXa^{+/-}$-K18-hACE2 mice and K18-hACE2 control mice were measured by q-PCR (a) and immunoblotting assay (b). N=4 or 5 biological independent mice in a. Data in b show two biologically independent mice in each group and are representative of two independent experiments with similar results. Data are presented as mean values ± SD and statistical analyses were performed by two-sided Student's t test (a). Source data are provided as a Source Data file.

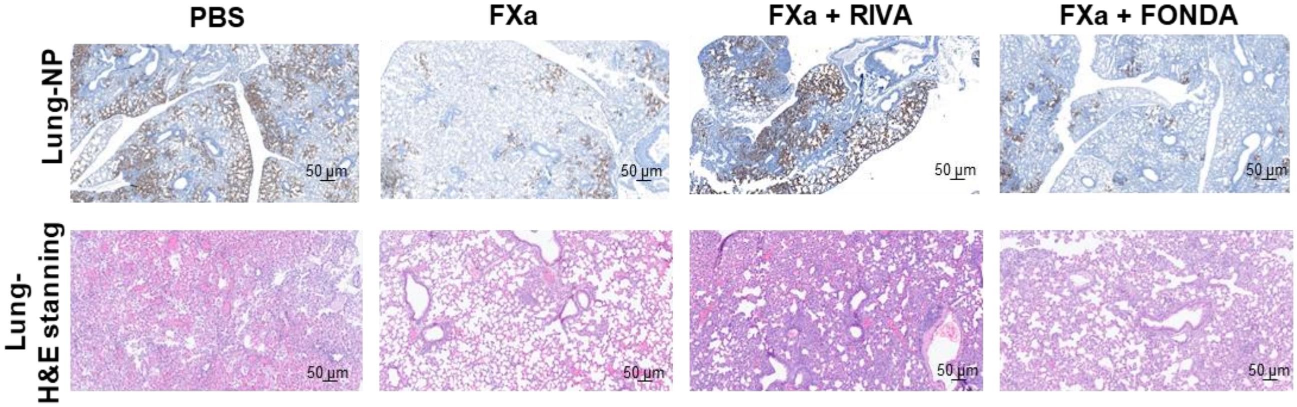

Supplementary Figure 10. IHC staining with an antibody against viral nuclear protein (NP) to detect SARS-CoV-2 in lungs of mice treated with FXa, FXa+RIVA, or FXa+FONDA. PBS served as the control. Pathological analysis of the lung of these mice as performed by H&E staining. All the mice were sacrificed at day 5 post infection to collect lung tissues for H&E staining and IHC with an anti-NP antibody. The data were repeated with 4 mice per group with similar results. Scale bar, 50 μm.

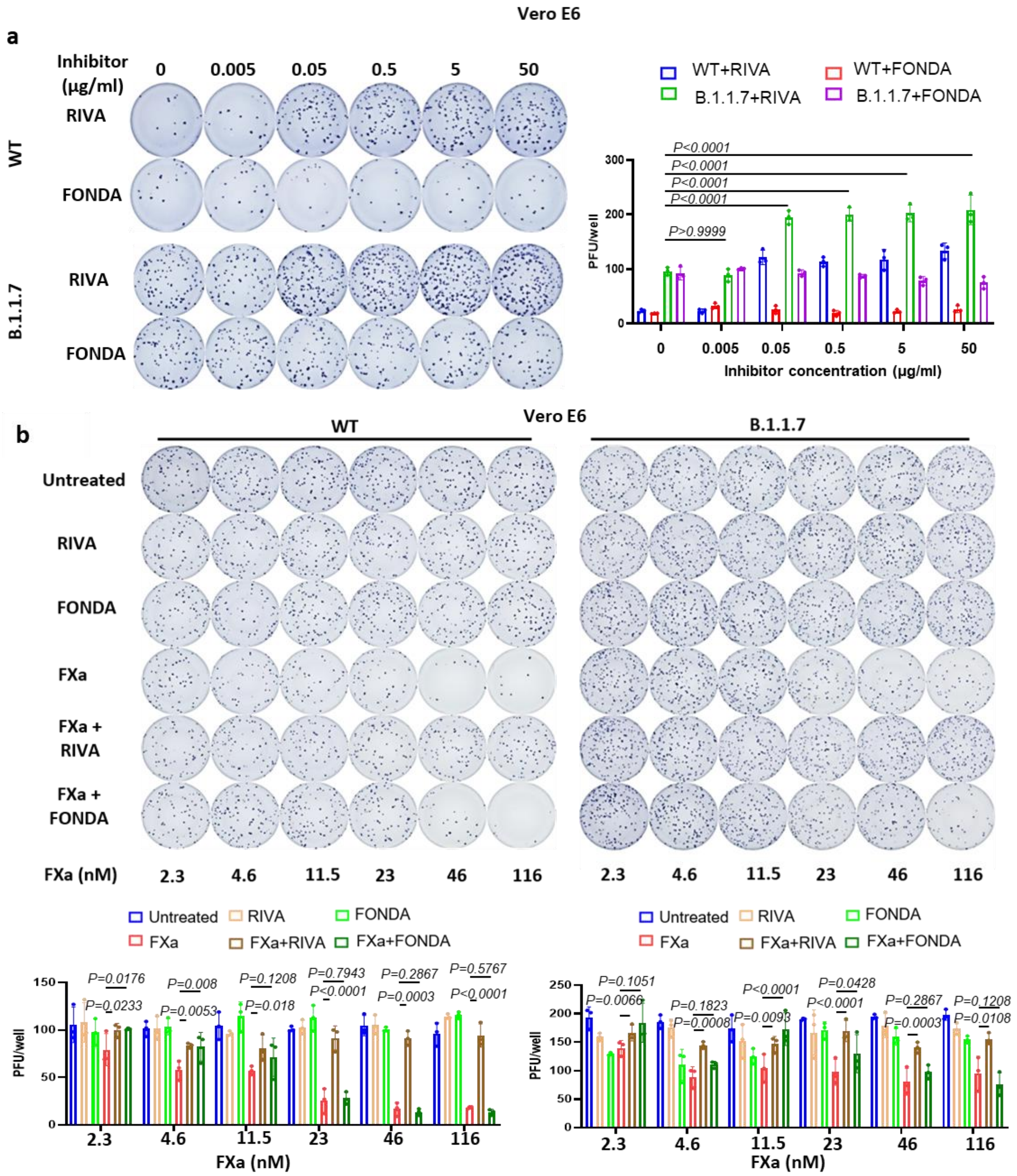

Supplementary Figure 11. Effect of various doses of RIVA or FONDA on infectivity of live WA1 SARS-CoV-2 or the B.1.1.7 variant pretreated with FXa in Vero E6 cells. **(a)** Vero E6 cells were infected with live WA1 SARS-CoV-2 or the B.1.1.7 variant that had been pretreated for 1 hr with different doses of RIVA or FONDA in the presence of FXa. At 24 hpi, viral infectivity was measured by immuno-plaque assay. A representative assay is shown on the left; summary data are on the right. **(b)** Vero E6 cells were infected with live WA1 SARS-CoV-2 or the B.1.1.7 variant pretreated with different doses of FXa in the presence of RIVA or FONDA. At 24 hpi, viral infectivity was measured by immuno-plaque assay (upper); summary data (lower). Data are presented as mean values ± SD and statistical analyses were performed with two-way ANOVA. N=3 independent experiments. Source data are provided as a Source Data file.

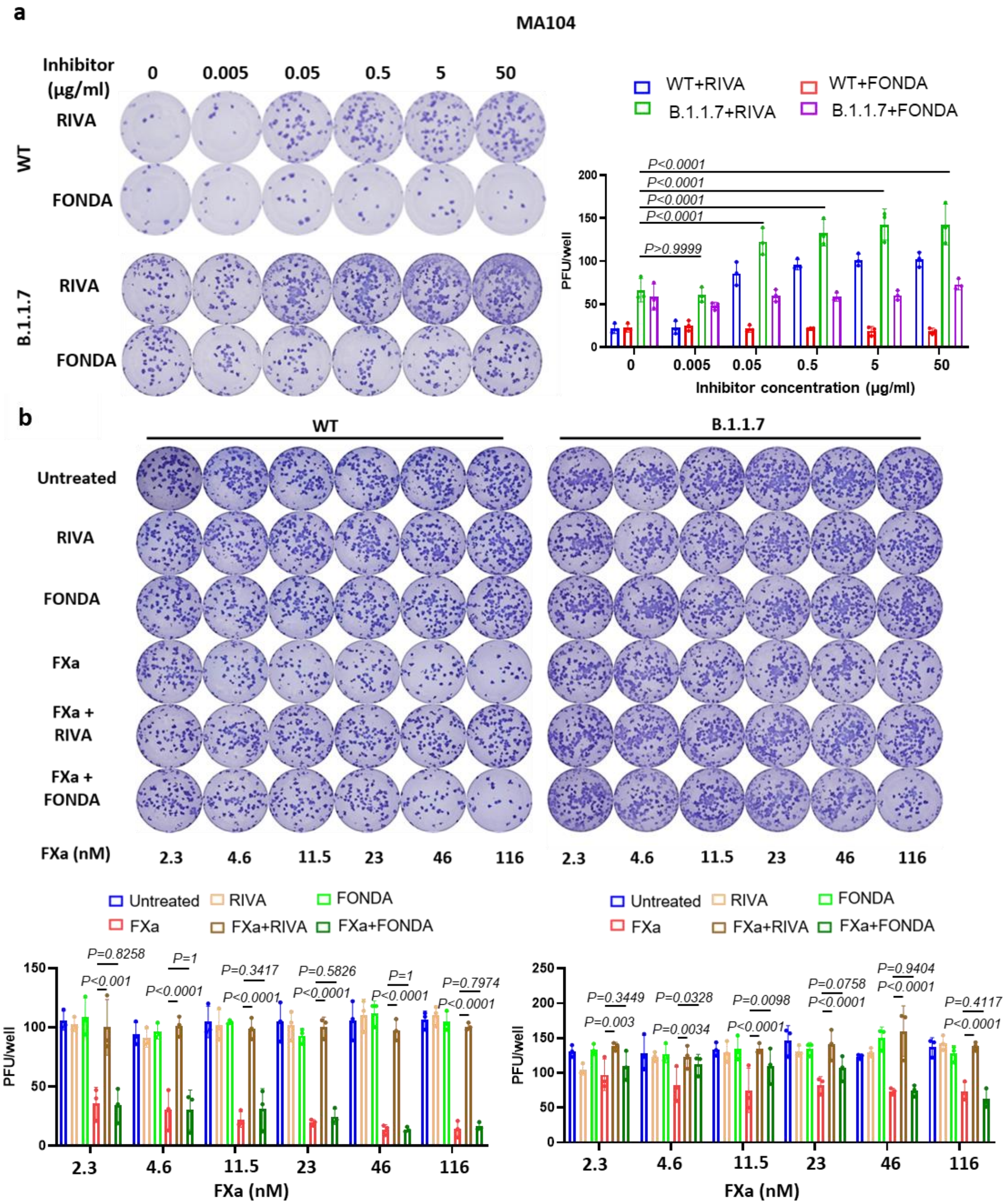

Supplementary Figure 12. Effect of various doses of RIVA or FONDA on infectivity of live WA1 SARS-CoV-2 or the B.1.1.7 variant pretreated with FXa in MA104 cells. **(a)** MA104 cells were infected with live WA1 SARS-CoV-2 or the B.1.1.7 variant that had been pretreated for 1 hr with different doses of RIVA or FONDA in the presence of FXa. At 24 hpi, viral infectivity was measured with an immuno-plaque assay. Left: a representative assay; right: summary data. **(b)** MA104 cells were infected with live WA1 SARS-CoV-2 or the B.1.1.7 variant. Both strains had been pretreated with different doses of FXa for 1 hr in the presence of RIVA or FONDA. At 24 hpi, viral infectivity was measured by immuno-plaque assay (upper). Summary data are presented in the lower panel. Data are presented as mean values ± SD and statistical analyses were performed with two-way ANOVA. N=3 independent experiments. Source data are provided as a Source Data file.

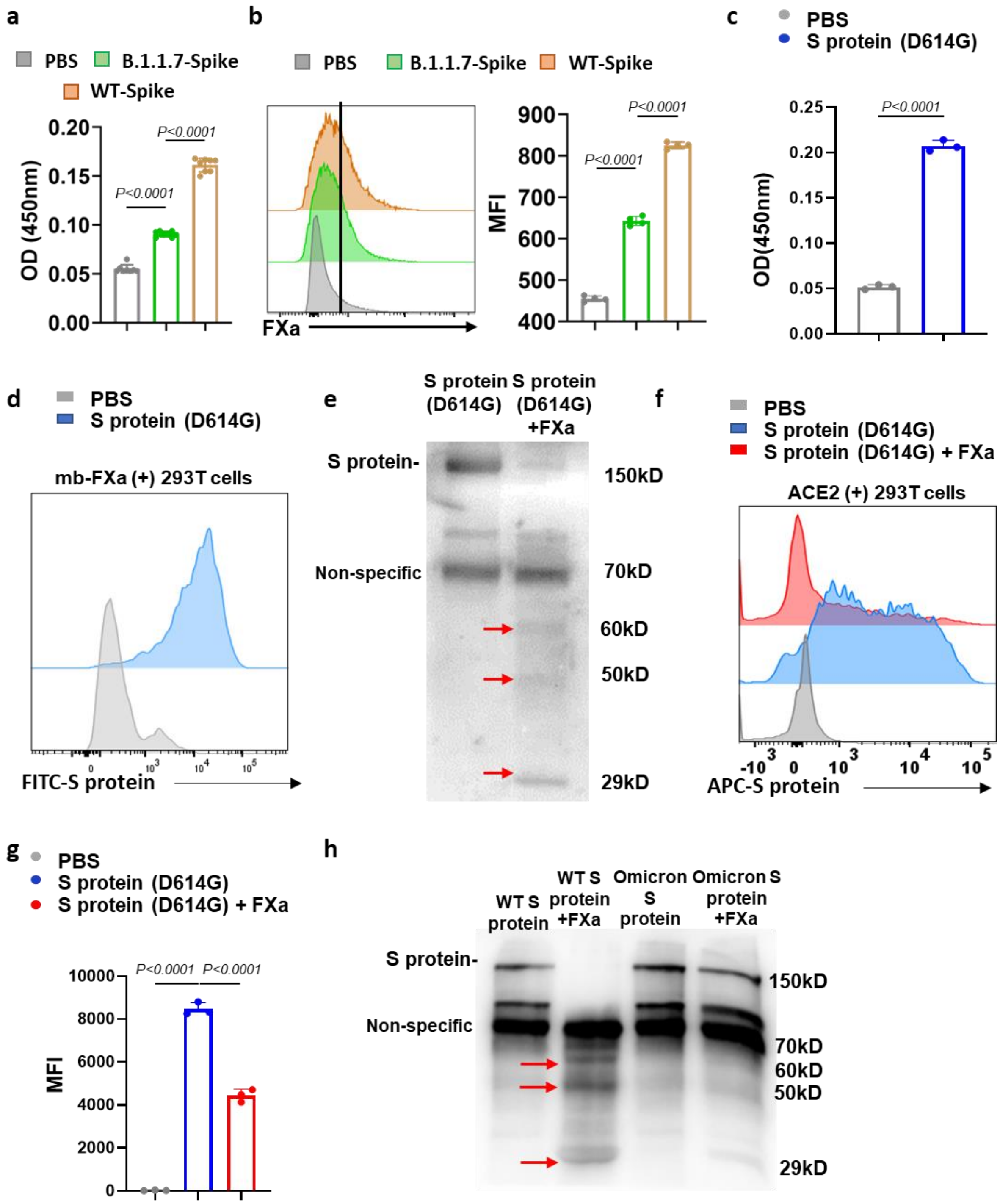

Supplementary Figure 13. Binding and cleavage of the mutant S protein by FXa. (**a**) Binding of FXa with WT WA1 S protein or B.1.1.7 variant S protein was assessed by ELISA. N=8 biological independent samples. (**b**) Binding of WT WA1 S protein or the B.1.1.7 S protein with FXa expressed on 293T cells, as assessed by flow cytometry. PBS was the control (representative flow cytometry histograms on the left; summary data on the right). N=4 independent experiments. (**c**) Binding affinity of FXa and the D614G S protein was measured by ELISA. N=3 independent samples. (**d**) Binding of the mutant S protein of the SARS-CoV-2 D614G variant with FXa expressed on 293T cells was assessed by flow cytometry. (**e**) Cleavage of the D614G S protein by FXa was assayed by immunoblotting with an anti-S protein antibody (40591-T62, Sino Biological). (**f** and **g**) Binding affinity of ACE2 and the D614G S protein pretreated or

not treated with FXa was measured by flow cytometry. N=3 independent experiments in (g). (**h**) Cleavage of WT WA1 S protein or Omicron S protein by FXa after 1-hour incubation was analyzed by immunoblotting using the same antibody in (e). All data are representative of at least three independent experiments. Data are presented as mean values ± SD and statistical analyses were performed two-sided Student's *t* test (c) and one-way ANOVA (a, b, g). Source data are provided as a Source Data file.

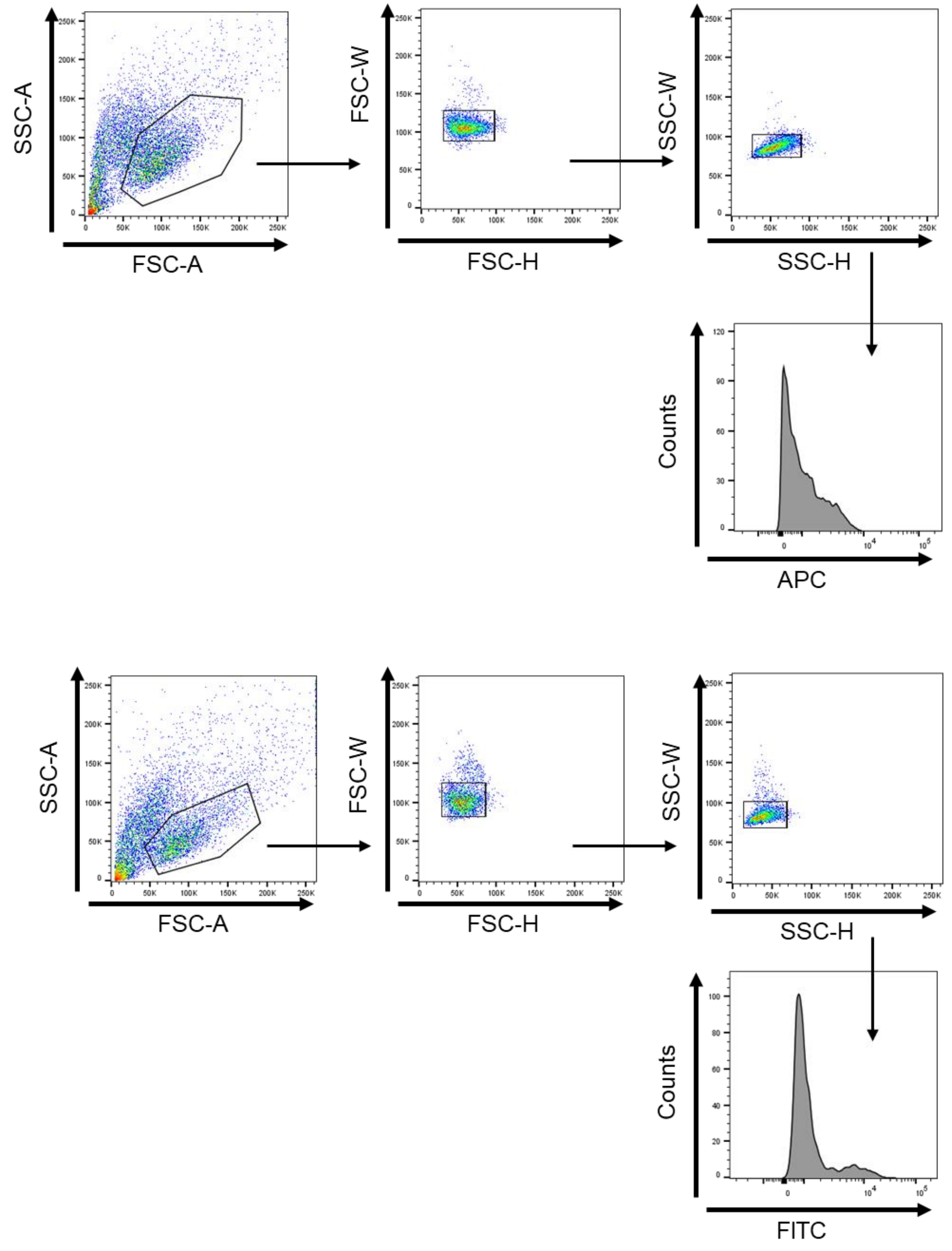

Supplementary figure 14. Flow cytometry gating strategy. Live cells were fist identified by low forward scatter (FSC) and low side scatter (SSC) gates. **(a)** To assay binding between the S protein and FXa, HEK293T cells expressing FXa were incubated with 10 µg/ml full-length S protein for 20 minutes at room temperature. Then the cells were washed twice and stained with anti-S protein antibody for 20 minutes at room temperature. After that, the cells were washed twice again and stained with an APC-labeled secondary antibody (111-605-045, Jackson ImmunoResearch). Median fluorescence intensity (MFI) of APC was used to determine the binding capacity of FXa to S protein (Figs. 3b, 3j, Supplementary Fig. 6a and Supplementary Fig. 13f). **(b)** To assay binding between the S protein–ACE2 complex and FXa, HEK293T cells stably expressing ACE2 protein were pre-incubated with the full-length S protein

for 1 hr, washed twice with media, and then incubated with or without FXa for 20 minutes at room temperature. Cells were then washed twice and incubated with an anti-S protein antibody for 20 minutes at room temperature. Then the cells were stained with an FITC-labeled secondary antibody (111-005-003, Jackson ImmunoResearch) and washed twice, followed by flow cytometry assay. MFI of FITC was used to determine the binding capacity of FXa to the S protein-ACE2 complex (Fig. 3d and Supplementary Fig. 13d).

Patient ID	age	sex	hospitalized	Treatment
COH001	60	M	no	none
COH003	58	M	no	none
COH004	68	F	no	none
COH006	45	M	yes	Leflunomide + Tocilizumab
COH007	64	F	no	oral steroids and inhalers
COH008	38	M	yes	Remdesivir, Tocilizumab, DAS181 and steroids
COH009	68	F	yes	Remdesivir
COH010	34	M	yes	Remdesivir
COH014	73	M	yes	Remdesivir, Toci, Plasma

Supplementary Table 1. Individual participant characteristics.
