# Supplementary material for: Factor Xa cleaves SARS-CoV-2 spike protein to block viral entry and infection
Source: Nat Commun. 2023 Apr 6;14:1936. doi: 10.1038/s41467-023-37336-9 (PMC10079155; doi:10.1038/s41467-023-37336-9)
Supplement: Supplementary file 2 — Description of Additional Supplementary Files [file 41467_2023_37336_MOESM2_ESM.pdf]

Description of Additional Supplementary Files

File Name: Supplementary Data 1
Description: List of proteins or peptides identified by LC-MS/MS for the non-specific band shown on the gel of cleavage assay
