# Supplementary material for: Factor Xa cleaves SARS-CoV-2 spike protein to block viral entry and infection
Source: Nat Commun. 2023 Apr 6;14:1936. doi: 10.1038/s41467-023-37336-9 (PMC10079155; doi:10.1038/s41467-023-37336-9)
Supplement: Supplementary file 4 — Reporting Summary [file 41467_2023_37336_MOESM4_ESM.pdf]

nature portfolio

Corresponding author(s): Prof. Michael A Caligiuri

Last updated by author(s): Feb 18, 2023

Reporting Summary

Nature Portfolio wishes to improve the reproducibility of the work that we publish. This form provides structure for consistency and transparency in reporting. For further information on Nature Portfolio policies, see our Editorial Policies and the Editorial Policy Checklist.

Statistics

For all statistical analyses, confirm that the following items are present in the figure legend, table legend, main text, or Methods section.

n/a	Confirmed	
☐	☒	The exact sample size (*n*) for each experimental group/condition, given as a discrete number and unit of measurement
☐	☒	A statement on whether measurements were taken from distinct samples or whether the same sample was measured repeatedly
☐	☒	The statistical test(s) used AND whether they are one- or two-sided<br>*Only common tests should be described solely by name; describe more complex techniques in the Methods section.*
☒	☐	A description of all covariates tested
☐	☒	A description of any assumptions or corrections, such as tests of normality and adjustment for multiple comparisons
☐	☒	A full description of the statistical parameters including central tendency (e.g. means) or other basic estimates (e.g. regression coefficient) AND variation (e.g. standard deviation) or associated estimates of uncertainty (e.g. confidence intervals)
☐	☒	For null hypothesis testing, the test statistic (e.g. *F*, *t*, *r*) with confidence intervals, effect sizes, degrees of freedom and *P* value noted<br>*Give P values as exact values whenever suitable.*
☒	☐	For Bayesian analysis, information on the choice of priors and Markov chain Monte Carlo settings
☒	☐	For hierarchical and complex designs, identification of the appropriate level for tests and full reporting of outcomes
☒	☐	Estimates of effect sizes (e.g. Cohen's *d*, Pearson's *r*), indicating how they were calculated

Our web collection on statistics for biologists contains articles on many of the points above.

Software and code

Policy information about availability of computer code

Data collection	No customized software was used; BD FACSDiva version 6 was used to collect flow cytometric data; Applied Biosystems StepOnePlus real-time PCR system and associated software were used to collect real-time PCR data; microscope data were collected using Zeiss ZEN and associated software (blue edition). Western blot data were collected by Odyssey CLx system. Additional information about software was described in the manuscript or available upon request.
Data analysis	No custom-made software was used for data analysis. Statistical analyses were performed using R3.4.0, SAS 9.4, Microsoft Excel 2016 or Graphpad Prism v.8. The Microscope images were analyzed using Zeiss ZEN (blue edition). Flow cytometry data were analyzed using Flowjo 7.6 or 10.0.Proteome Discoverer SEQUEST (version 2.5, Thermo Scientific) was used to analyze liquid chromatography mass spectrometry data.

For manuscripts utilizing custom algorithms or software that are central to the research but not yet described in published literature, software must be made available to editors and reviewers. We strongly encourage code deposition in a community repository (e.g. GitHub). See the Nature Portfolio guidelines for submitting code & software for further information.

Data

Policy information about availability of data

All manuscripts must include a data availability statement. This statement should provide the following information, where applicable:

- Accession codes, unique identifiers, or web links for publicly available datasets
- A description of any restrictions on data availability
- For clinical datasets or third party data, please ensure that the statement adheres to our policy

Uniprot SARS-COV-2 Spike database (https://covid-19.uniprot.org/uniprotkb/P0DTC2) was used as a reference for liquid chromatography mass spectrometry data. Source data for Figs. 1–7 and Extended Data Figs. 1–13 have been provided as Source Data files. All other data supporting the findings of this study are available from the corresponding author upon reasonable request.

Human research participants

Policy information about studies involving human research participants and Sex and Gender in Research.

Reporting on sex and gender	The human research participant is not a sex/gender related research. No sex-based analysis was performed.
Population characteristics	Peripheral blood of COVID-19 patients and healthy donors were collected regardless of specific age, gender or other information.
Recruitment	Healthy donors donated the blood in Michael Amini Transfusion Medicine Center of City of Hope National Medical Center. COVID-19 patients were recruited in Division of Infectious Diseases, Department of Medicine, City of Hope National Medical Center.
Ethics oversight	Peripheral blood of healthy donors or COVID-19 patients was collected after written informed consent under protocols approved by the City of Hope Institutional Review Board.

Note that full information on the approval of the study protocol must also be provided in the manuscript.

Field-specific reporting

Please select the one below that is the best fit for your research. If you are not sure, read the appropriate sections before making your selection.

☒ Life sciences ☐ Behavioural & social sciences ☐ Ecological, evolutionary & environmental sciences

For a reference copy of the document with all sections, see nature.com/documents/nr-reporting-summary-flat.pdf

Life sciences study design

All studies must disclose on these points even when the disclosure is negative.

Sample size	No statistical method was used to predetermine the sample size. Sample sizes in this study were used based on previous experience that showed significance (PMID: 30475349, 34625564, and 34006525).For in vitro studies, the experiments were repeated at least 3 times with samples size at least 3 to control technical variations. For survival studies, at least 5 animals for each group were used to obtain informative results.
Data exclusions	No data were excluded for all figures.
Replication	All experiments were reliably reproduced and results are represented as mean +/-SD as appropriate. All experiments were repeated with at least three donors or three independent times. One-way ANOVA model was utilized to compare three or more conditions. For data with repeated measures from the same donor, linear mixed model was utilized to account for the variance-covariance structure due to repeated measures. When needed, P values were adjusted for multiple comparisons using the Bonferroni method procedure. A P value of 0.05 or less was considered statistically significant, which is described in the methods section of the main text.
Randomization	Animals were randomly grouped for in vivo experiments.
Blinding	For the survival study, the investigators who collected the data were blinded to the treatment. No blinding was used in vitro experiments. The majority of data collection was quantifiable and blinding would not change any bias in data collected.

Reporting for specific materials, systems and methods

We require information from authors about some types of materials, experimental systems and methods used in many studies. Here, indicate whether each material, system or method listed is relevant to your study. If you are not sure if a list item applies to your research, read the appropriate section before selecting a response.

Materials & experimental systems

n/a	Involved in the study
☐	☒ Antibodies
☐	☒ Eukaryotic cell lines
☒	☐ Palaeontology and archaeology
☐	☒ Animals and other organisms
☒	☐ Clinical data
☒	☐ Dual use research of concern

Methods

n/a	Involved in the study
☒	☐ ChIP-seq
☐	☒ Flow cytometry
☒	☐ MRI-based neuroimaging

Antibodies

Antibodies used

Antibodies used for flow cytometry:
Anti-SARS-CoV-2 spike glycoprotein antibody (CAT:ab272504, LOT:GR3345969-3, Abcam); using dilution:1:1000.
Alexa Fluor® 647 AffiniPure Goat Anti-Rabbit IgG (H+L) (CAT:111-605-045, LOT:140958, Jackson ImmunoResearch); using dilution:1:1000.
Alexa Fluor® 488 AffiniPure Donkey Anti-Rabbit IgG (H+L) (CAT:711-545-152, LOT:144217, Jackson ImmunoResearch); using dilution:1:1000.

Antibodies used for immunoprecipitation:
Anti-SARS-CoV-2 spike glycoprotein antibody (CAT:ab272504, LOT:GR3345969-3,Abcam), using dilution:1:1000.

Antibodies used for immunohistochemistry staining:
Factor X Rabbit anti-Human, Mouse, Polyclonal, Invitrogen™ (CAT:PIPA529118, Invitrogen); using dilution:1:500.
Recombinant Anti-Furin antibody [EPR14674] (CAT:ab183495, Abcam); using dilution:1:500.
Recombinant Anti-Trypsin antibody [EPR19498] (CAT:ab200997, Abcam); using dilution:1:500.
Polyclonal Rabbit anti-Mouse PLG / Plasmin / Plasminogen Antibody (IHC, WB) (CAT:LS-C150813-1, LSBio); using dilution:1:500.
Anti-Thrombin antibody [5G9] (CAT:ab17199, Abcam); using dilution:1:500.
SARS Nucleocapsid Protein Antibody (CAT:NB100-56576, Novus); using dilution:1:500.
Goat Anti-Rabbit IgG H&L (HRP) (CAT:ab6721, Abcam); using dilution:1:500.

Antibodies used for immunoblotting assay:
SARS-CoV-2 (2019-nCoV) Spike Antibody, Rabbit PAb, Antigen Affinity Purified (CAT: 40591-T62, Sino Biological); using dilution:1:1000.
SARS-CoV-2 Spike RBD Antibody (clone: 1034522) (CAT:MAB10540-100, R&D); using dilution:1:1000.
SARS/SARS-CoV-2 Spike Protein S2 Antibody (1A9) (CAT:MA5-35946, Invitrogen); using dilution:1:1000.

Validation

Antibodies used for flow cytometry:
Anti-SARS-CoV-2 spike glycoprotein antibody (CAT:ab272504, LOT:GR3345969-3,Abcam).Please see the manufacturer's website link for application. https://www.abcam.com/sars-cov-2-spike-glycoprotein-antibody-coronavirus-ab272504.html
Alexa Fluor® 647 AffiniPure Goat Anti-Rabbit IgG (H+L) (CAT:111-605-045, LOT:140958, Jackson ImmunoResearch).Please see the manufacturer's website link for application. https://www.jacksonimmuno.com/catalog/products/111-605-045
Alexa Fluor® 488 AffiniPure Donkey Anti-Rabbit IgG (H+L) (CAT:711-545-152, LOT:144217, Jackson ImmunoResearch).Please see the manufacturer's website link for application. https://www.jacksonimmuno.com/catalog/products/711-545-152

Antibodies used for immunoprecipitation:
Anti-SARS-CoV-2 spike glycoprotein antibody (CAT:ab272504, LOT:GR3345969-3,Abcam).Please see the manufacturer's website link for application. https://www.abcam.com/sars-cov-2-spike-glycoprotein-antibody-coronavirus-ab272504.html

Antibodies used for Immunohistochemistry assay:
Factor X Rabbit anti-Human, Mouse, Polyclonal, Invitrogen™ (PIPA529118, Invitrogen), Please see the manufacturer's website link for application. https://www.fishersci.com/shop/products/anti-factor-x-polyclonal-pa5-29118/PIPA529118
Recombinant Anti-Furin antibody [EPR14674] (ab183495, Abcam), Please see the manufacturer's website link for application. https://www.abcam.com/furin-antibody-epr14674-ab183495.html
Recombinant Anti-Trypsin antibody [EPR19498] (ab200997, Abcam). Please see the manufacturer's website link for application. https://www.abcam.com/trypsin-antibody-epr19498-ab200997.html
Polyclonal Rabbit anti-Mouse PLG / Plasmin / Plasminogen Antibody (IHC, WB) (LS-C150813-1, LSBio). Please see the manufacturer's website link for application. https://www.lsbio.com/antibodies/plg-antibody-plasmin-antibody-plasminogen-antibody-elisa-ihc-wb-western-ls-c150813/157056
Anti-Thrombin antibody [5G9] (ab17199, Abcam).Please see the manufacturer's website link for application. https://www.abcam.com/thrombin-antibody-5g9-ab17199.html
SARS Nucleocapsid Protein Antibody (NB100-56576, Novus).Please see the manufacturer's website link for application. https://www.novusbio.com/products/sars-nucleocapsid-protein-antibody_nb100-56576?gclid=Cj0KCQjwzYGGBhCTARIsAHdMTQxP6VKJjSpqXM4cBNsWnuGDdRA-ttVgvbX6JnWb3BNVRRe5J4FM_aAaAszfEALw_wcB
Goat Anti-Rabbit IgG H&L (HRP) (CAT:ab6721, Abcam).Please see the manufacturer's website link for application. https://www.abcam.com/goat-rabbit-igg-hl-hrp-ab6721.html

Eukaryotic cell lines

Policy information about cell lines and Sex and Gender in Research

Cell line source(s)	Vero cells were obtained from the laboratory of E. Antonio Chiocca. MA104 cells were obtained from the laboratory of Dr. Sean P.J. Whelan. Human embryonic kidney-derived HEK293T cells, Vero E6 cells, Calu-3 cells, human lung cancer cell line A549, and Chinese hamster ovary (CHO) cells were purchased from ATCC.
Authentication	None of the cell lines used were authenticated.
Mycoplasma contamination	The cell lines were tested for mycoplasma contamination. All the cells used in this study were mycoplasma negative.
Commonly misidentified lines (See ICLAC register)	No commonly misidentified cell lines were used.

Animals and other research organisms

Policy information about studies involving animals; ARRIVE guidelines recommended for reporting animal research, and Sex and Gender in Research

Laboratory animals	Six- to eight-week-old K18-hACE2 mice were purchased from Jackson Laboratories (Bar Harbor, Maine). FXa knockout C57BL/6J mice were generated at Biocytogen (Wakefield, Massachusetts). All mice with six- to eight-week-old were housed in City of Hope Animal Facility or Northern Arizona University Animal Facility with light cycle: A 12-light/12-dark cycle and temperatures of 65-75°F (- 18-23° C) with 40-60% humidity.
Wild animals	No wild animals were used in this study.
Reporting on sex	Both female and male K18-hACE2 mice and FXa knockout C57BL/6J were used in this study. No sex-based analysis was performed.
Field-collected samples	No field-collected samples were used.
Ethics oversight	Experiments and handling of mice were conducted under federal, state, and local guidelines and with approvals from the Northern Arizona University and City of Hope Animal Care and Use Committees.

Note that full information on the approval of the study protocol must also be provided in the manuscript.

Flow Cytometry

Plots

Confirm that:

☒ The axis labels state the marker and fluorochrome used (e.g. CD4-FITC).

☒ The axis scales are clearly visible. Include numbers along axes only for bottom left plot of group (a 'group' is an analysis of identical markers).

☒ All plots are contour plots with outliers or pseudocolor plots.

☒ A numerical value for number of cells or percentage (with statistics) is provided.

Methodology

Sample preparation	For the S protein and FXa binding assays, HEK293T cells expressing FXa were incubated with 10 µg/ml full-length S protein for 20 minutes at room temperature. Then the cells were washed twice and stained with anti-S protein antibody for 20 minutes at room temperature. After that the cells were washed twice and stained with followed by staining with a APC-labeled secondary antibody (111-605-045, Jackson ImmunoResearch). Median of mean fluorescence intensity (MFI) of APC was used to determine S protein binding capacity of FXa.<br>For the FXa and wild type S protein or B.1.1.7 variant S protein binding assays, HEK293T cells expressing wild type S protein or B.1.1.7 variant S protein were incubated with 10 µg/ml FXa-Fc for 20 minutes at room temperature. After that the cells were washed twice and stained with followed by staining with a APC-labeled secondary antibody (111-605-045, Jackson ImmunoResearch). Median of mean fluorescence intensity (MFI) of APC was used to determine wild type S protein or B.1.1.7 variant S protein binding capacity of FXa.<br>For the S protein-ACE2 complex and FXa binding assays,HEK293T cells stably expressing ACE2 protein were incubated with full-length S protein or FXa-pretreated full-length S protein for 20 minutes at room temperature. Cells were then washed and incubated with an anti-S protein antibody for 20 minutes at room temperature, followed by staining with an FITC-labeled secondary antibody (111-005-003, Jackson ImmunoResearch). Median of mean fluorescence intensity (MFI) of FITC was used to determine S protein-ACE2 complex binding capacity of FXa.
Instrument	Fortessa X20 flow cytometer (BD Biosciences) was used to collect flow cytometric data.
Software	BD FACSDiva and FlowJo 7.6 & 10.0 were used to collect and analyze the flow cytometric data, respectively.

Cell population abundance	The purity of sorted cells was over 99%.
Gating strategy	For the S protein and FXa binding assays, live cells were further gated on FSC-A/SSC-A, FSC-H/FSC-W, and then SSC-H/SSC-W events. APC channel was used to check surface expression levels of S protein or FXa.<br>For the S protein-ACE2 complex and FXa binding assays, live cells were further gated on FSC-A/SSC-A, FSC-H/FSC-W, and then SSC-H/SSC-W events. FITC channel was used to check surface expression levels of S protein.

☒ Tick this box to confirm that a figure exemplifying the gating strategy is provided in the Supplementary Information.
